# Gene Transcript Alterations in the Spinal Cord, Anterior Cingulate Cortex, and Amygdala in Mice Following Peripheral Nerve Injury

**DOI:** 10.3389/fcell.2021.634810

**Published:** 2021-04-07

**Authors:** Songxue Su, Mengqi Li, Di Wu, Jing Cao, Xiuhua Ren, Yuan-Xiang Tao, Weidong Zang

**Affiliations:** ^1^Department of Anatomy, College of Basic Medicine, Zhengzhou University, Zhengzhou, China; ^2^Neuroscience Research Institute, Zhengzhou University Academy of Medical Sciences, Zhengzhou, China; ^3^Department of Anesthesiology, Pain and Perioperative Medicine, The First Affiliated Hospital of Zhengzhou University, Zhengzhou, China; ^4^Department of Bioinformatics, College of Life Sciences, Zhengzhou University, Zhengzhou, China; ^5^Department of Anesthesiology, Rutgers New Jersey Medical School, The State University of New Jersey, Newark, NJ, United States

**Keywords:** SNL (spinal nerve ligation), neuropathic pain, emotion disorder, spinal cord (SC), anterior cingulate cortex (ACC), amygdala (AMY), RNA sequencing, differentialli expressed genes (DEGs)

## Abstract

Chronic neuropathic pain caused by nerve damage is a most common clinical symptom, often accompanied by anxiety- and depression-like symptoms. Current treatments are very limited at least in part due to incompletely understanding mechanisms underlying this disorder. Changes in gene expression in the dorsal root ganglion (DRG) have been acknowledged to implicate in neuropathic pain genesis, but how peripheral nerve injury alters the gene expression in other pain-associated regions remains elusive. The present study carried out strand-specific next-generation RNA sequencing with a higher sequencing depth and observed the changes in whole transcriptomes in the spinal cord (SC), anterior cingulate cortex (ACC), and amygdala (AMY) following unilateral fourth lumbar spinal nerve ligation (SNL). In addition to providing novel transcriptome profiles of long non-coding RNAs (lncRNAs) and mRNAs, we identified pain- and emotion-related differentially expressed genes (DEGs) and revealed that numbers of these DEGs displayed a high correlation to neuroinflammation and apoptosis. Consistently, functional analyses showed that the most significant enriched biological processes of the upregulated mRNAs were involved in the immune system process, apoptotic process, defense response, inflammation response, and sensory perception of pain across three regions. Moreover, the comparisons of pain-, anxiety-, and depression-related DEGs among three regions present a particular molecular map among the spinal cord and supraspinal structures and indicate the region-dependent and region-independent alterations of gene expression after nerve injury. Our study provides a resource for gene transcript expression patterns in three distinct pain-related regions after peripheral nerve injury. Our findings suggest that neuroinflammation and apoptosis are important pathogenic mechanisms underlying neuropathic pain and that some DEGs might be promising therapeutic targets.

## Introduction

Neuropathic pain characterized by a broad range of sensory, cognitive, and emotional dysfunction is a complex and debilitating public health problem that affects about 7–10% of the gross population worldwide (van Hecke et al., 2014; Bushnell et al., 2015; Colloca et al., 2017). Clinical and preclinical investigations have observed clusters of behavioral symptoms including spontaneous pain, evoked nociceptive behaviors, pain aversiveness, anxiety, and depression in neuropathic pain rodents and patients (Seminowicz et al., 2009; LaCroix-Fralish et al., 2011). These abnormal behaviors may involve the changes in the activities of nociceptive neurons and the emergence of the new pathological processes and signaling pathways (von Hehn et al., 2012; Guo et al., 2016). However, current treatments have not yielded satisfactory results. Opioids and non-steroidal anti-inflammatory drugs (NSAID) are considered effective approaches to relieve these symptoms, but the efficacy should be re-appraised because of possible safety concerns (Finnerup et al., 2015; Jones et al., 2018). Therefore, identifying the gene expression profiles and gene interactions in pain-associated regions is essential for understanding the pathogenesis under neuropathic pain and developing novel therapeutic strategies to improve the treatment outcomes (Samuel and Farsides, 2017).

Abnormal changes in neural activity and plasticity arising from tissue or nerve injury contribute to pain hypersensitivity (von Hehn et al., 2012). The spinal cord is responsible for receiving information from nociceptors and projecting to the brain and plays a major role in integrating and modulating nociceptive signals (Kuner, 2010). Studies have reported that brain regions anterior cingulate cortex (ACC) and amygdala (AMY) are important areas in pain sensation and involved in the interpretation and assessment of the affective and emotional components of pain (Gao et al., 2004; Phelps and LeDoux, 2005; Cao et al., 2009; Navratilova et al., 2015; Neugebauer, 2015). The lesion of ACC and AMY was documented to inhibit the conditioned place aversion of formalin (Gao et al., 2004; Cao et al., 2009). Increasing evidence indicates that cellular and molecular adaptations within these two regions appear under chronic stress and chronic pain conditions (Simons et al., 2014; Ji et al., 2017; Sellmeijer et al., 2018; Navratilova et al., 2019). However, gene expression patterns in these two areas after nerve injury have not been examined. Moreover, previous studies identified a large amount of differentially expressed genes (DEGs) using gene microarrays and RNA sequencing in neuropathic pain (Jiang et al., 2015; Wu et al., 2016; Descalzi et al., 2017; Zhou et al., 2017), but these studies did not provide a full comparison among distinct pain-associated regions as most of them focused on only one region. Thus, it is imperative to have a comprehensive understanding by sequencing and comparing the gene expression patterns in different pain-associated regions.

To this end, in the present study, we carried out a more thorough analysis of gene expression alterations after nerve injury by examining the DEGs in the spinal cord, ACC, and AMY. A mouse model of L4 spinal nerve ligation (SNL) and the next-generation RNA sequencing with a higher sequencing depth were conducted. Our results revealed the unique transcriptional profiles across three regions responding to peripheral nerve injury and the significant overlapping effects implicating in biological functions and signaling pathways despite some differences. Functional analysis demonstrated that the pain-, anxiety-, and depression-related DEGs were closely associated with neuroinflammation and apoptosis. The conspicuous overlap of pain-, anxiety-, and depression-related DEGs among three regions illustrates some conservative changes in the transcriptome independent of regions. Therefore, our findings may bring some useful information and novel insights into the molecular mechanism that will lead to a new direction for further studies and a potential development of clinical analgesic medications.

## Methods

### Animal Preparation

Eight-week-old male C57BL/6 mice (25–28 g) were purchased from the Animal Experiment Center (Zhengzhou, Henan, China) and housed in the central animal facility under a standard 12-h light/12-h dark cycle with food and water *ad libitum*. The mice were kept for at least 7 days before the experiments. The procedures for the care and use of animals were approved by the Animal Care and Use Committee of Zhengzhou University and performed under the guidelines of the International Association for the Study of Pain.

### L4 SNL-Induced Neuropathic Pain Model

Mice underwent unilateral L4 SNL (the fourth lumbar L4 spinal nerve) as previously described (Wu et al., 2016; Zhou et al., 2017; Sun et al., 2019). Briefly, animals were anesthetized with isoflurane and the L4 spinal nerve was exposed through the removal of the L5 transverse process. After the exposure and isolation of the L4 spinal nerve, a tight ligation with 7–0 silk thread was made and the nerve was transected distal to the ligature. The surgical procedure for the sham group was identical to that of the SNL group, except that the spinal nerves were not transected or ligated.

### Behavioral Testing

Animals were habituated to the testing room with a stable temperature for at least 1 day before behavioral measurements.

#### Von Frey Filament Testing

Mice were put in individual Plexiglas chambers elevated on the mental mesh screen, and 30 min was allowed for adapting to the environment before the testing. The calibrated von Frey filaments (0.07 g and 0.4 g) were used to stimulate the plantar surface of each hind paw for 1 second. Each application represented one trial, and 10 trials were performed for each hind paw. The times were recorded when the animal exhibited a response (withdrawal, flicking, flinching, or licking) to the stimulation for each set of 10 trials. The paw withdrawal frequencies in 10 trials were calculated to evaluate the mechanical sensitivity (Sun et al., 2019; Li Y. et al., 2020).

### Hargreaves Assay

Animals were placed in individual Plexiglas chambers on a glass plate and acclimated for 30 min before the testing. The light beam in a Model 336 Analgesia Meter (UGO BASILE S.R.L., Italy) was applied. The radiant heat stimulus was turned off automatically when the animal displayed paw withdrew. The duration between the light application and paw withdrawing was considered as the paw withdrawal latency (PWL). The test for each paw was repeated three to five times at 5-min intervals. A cutoff of 20 s was set to avoid tissue damage (Sun et al., 2019; Li Y. et al., 2020).

### Cold Plate Assay

Animals were placed in a chamber on the cold plate with the temperature at 0°C, which was monitored continuously by a thermometer. The duration between the placement and the first sign of mouse jumping and/or flinching was recorded as the paw withdrawal latency (PWL) to noxious cold stimuli. Each test was repeated three times at 10-min intervals for the ipsilateral hind paw. The cutoff of 20 s was set to avoid tissue damage (Sun et al., 2019; Li Y. et al., 2020).

### Conditioned Place Aversion (CPA) Testing

The CPA test was carried out as previously described with minor modifications (Guo et al., 2016; Wu et al., 2019). Briefly, the CPA apparatus consisted of two Plexiglas chambers (15 cm × 15 cm × 15 cm) elevated on the mental mesh screen with a removable board in the middle (15 cm × 15 cm). The experimental process included three distinct sessions: a preconditioning session, a conditioning session, and a postconditioning (testing) session with a duration of 10 min in each session. Before the testing, animals experienced the habituation to the apparatus for at least three consecutive days. Six days post-surgery, animals were allowed to freely explore two chambers for 10 min in the preconditioning session. The amount of the time spent in each chamber was recorded. The mice with a strong initial bias (time spent in one chamber > 500 s) were excluded from the study. In the conditioning session, mice were trained in chambers paired with two von Frey filaments. Low-force (0.07 g) von Frey filament was paired with the non-preferred chamber, in which animals spent less time at the preconditioning session. Medium force (0.4 g) was paired with the preferred chamber in which animals stayed longer at the preconditioning session. During the 10-min training, von Frey filaments were used to stimulate ipsilateral hind paw of sham or SNL mice in the corresponding chamber for 5 min with 10-s intervals. Finally, in the postconditioning (testing) session, mice were allowed free access to both chambers. The duration of time that each mouse spent in each chamber was then recorded for 10 min. Results were presented as “Time in chamber” and “CPA score” that was calculated by the time recorded in pre-condition minus the time recorded in the post-condition.

### Tissue Collection and RNA Extraction

Briefly, two groups of mice (SNL and Sham) with three biological replicates were used. Unilateral punches were taken from the SC, ACC, and AMY, respectively. The punches per region were pooled from three mice per sample. A total of nine animals per treatment group were needed. Total RNA was extracted using the miRNeasy kit with on-column digestion of genomic DNA (QIAGEN, Valencia, CA, United States) according to the manufacturer’s instructions. RNA was purified with RNeasy Micro Kit 50 (cat. 74004, Qiagen), and the concentration was measured using the NanoDrop 2000 Spectrophotometer (Thermo Scientific, Wilmington, DE). Sample quality was evaluated with the ratios of A260/280 (1.97∼2.08) and RNA integrity numbers (RIN, 7.5∼8.4) as demonstrated by An Agilent 2100 Bioanalyzer (Agilent Technologies, Santa Clara, CA).

### RNA Sequencing

The total RNA (1.0 μg/sample) was subjected to rRNA depletion by Ribo-Zero rRNA Removal (Human/Mouse/Rat) Kit (Illumina, San Diego, CA, United States). Strand-specific RNA libraries were prepared using TruSeq Stranded Total RNA Sample Preparation Kit (Illumina) without poly-A selection. All assays were performed according to the manufacturer’s instructions. RNA-seq was performed on the Illumina Nova6000 plate High Output Model (Illumina, San Diego, United States) (Hrdlickova et al., 2017), in a 2 × 150-bp paired-end configuration, with a total of more than 2,666 M reads per lane (at least 50 M reads per sample).

### Bioinformatics Analysis

The samples from the SC, ACC, and AMY were subjected to multiplexing, sequencing, and differential gene expression analysis such as transcript expression analysis and ncRNA expression analysis. Briefly, Trimmomatic 0.32 was used to trim the sequences (minimal length 50 base pairs, leading and trailing Phred Q 30) for the quality first. The resulting sequencing data were then mapped to the musculus genome sequence version GRCm38.72 downloaded from ENSEMBLE. Gene hit counts and reads per kilobase per million (RPKM) were calculated for each gene to determine the expression levels within the CLCbio software environment (CLC Genomics Workbench 7.0.2, CLC genomics Server). Mapped reads were visualized on the UCSC browser using bigwig files converted from bam files. The significant differentially expressed (DE) mRNAs were defined using a cutoff of *P* < 0.05 and fold change ≥ 1.74 (log2(± 0.8)) to include more DEGs for the subsequent analyses, such as Go term and KEGG pathway analysis, to get more useful information, especially for the functional analysis and comparisons among three distinct regions. The heatmaps were generated via OmicShare (^1^ GENE *DENOVO*). The function of DE mRNAs was analyzed using the downloaded Gene Cards database^2^ and Comparative Toxicogenomics Database^3^. The DE mRNAs in the SC were mapped to pain-related genes (refer to pain/itch/touch/thermal/chemical related genes), and the DEGs in the ACC and AMY were aligned to pain- and emotion-related genes (refer to anxiety/depression-related genes). Moreover, the DEGs were compared with the genes related to neuroinflammation (inflammation and immunity) and apoptosis. A Venn diagram was also employed for the DEG comparisons^4^.

### Quantitative Real-Time RT-PCR

The RNA-sequence results were verified by q-RT-PCR. Total RNA was extracted from tissues as described above, treated using DNase I (New England Biolabs, Ipswich, MA, United States), and finally reversely transcribed with the Revert Aid First Strand cDNA Synthesis Kit (Thermo) according to the manufacturer’s instructions either oligo (dT) primers or specific RT primers. A template (2 μL) was used for the amplification by real-time PCR with primers as shown in Table 1 (Sangon Biotech, Shanghai, China). Each sample was run in triplicate in a 20-μL volume for the reaction containing 250 nM forward and reverse primers, 10 μL Thermo Scientific Maxima SYBR Green qPCR Master Mix (2×; Thermo Scientific Maxima SYBR Green qPCR Master Mix, Rox solution provided), and 20 ng total cDNA. Reactions were implemented in a 7500 Fast Real-Time PCR Detection System (Applied Biosystems, United States). The cycle parameters were set as follows: an initial 3-min incubation at 95°C, followed by 40 cycles of 95°C for 10 s, 60°C for 30 s, and 72°C for 30 s. Ratios of ipsilateral-side mRNA levels to contralateral-side mRNA levels were calculated using the Δ Ct method (2^−ΔΔCt^). All data were normalized to *Tuba1*α (Wu et al., 2016), which was identified as stable in mice after nerve injury.

**TABLE 1:** Primers for RT-qPCR.

Primer names	Sequences
*Tuba1a-F*	GTG CAT CTC CAT CCA TGT TG
*Tuba1a-R*	GTG GGT TCC AGG TCT ACG AA
*Cmklr1-F*	TGC ATG AAC CCC ATT CTG TA
*Cmklr1-R*	TGG TGA AGC TCC TGT GAC TG
*Adrb3-F*	ACA GGA ATG CCA CTC CAA TC
*Adrb3-R*	TTA GCC ACA ACG AAC ACT CG
*P2ry12-F*	CCT GGG GTT GAT AAC CAT TG
*P2ry12-R*	AAC ATG AAG GCC CAG ATG AC
*Kcnma1-F*	CCC AAT AGA ATC CTG CCA GA
*Kcnma1-R*	ATC GTT GGC TGC AAT AAA CC
*Kcnk18-F*	AGG AAG CCA TCC CTC AGA TT
*Kcnk18-R*	CAG GAG TTG CTC CTC TCC AC
*Mir9-3hg-F*	CAC ATG CCT AGA CAG GAG CA
*Mir9-3hg-R*	ACT ATC CAG CCA GTG GGA TG
*Miat-F*	AAA CCT GAG TCC TGG TGT GG
*Miat-R*	AAA AAC AGG TGG CCA AAG TG
*Pantr1-F*	GGA GAG GGA CAG AGT GCC TA
*Pantr1-R*	AAC CCC TGG ATA GGA CCA AC

*RT-qPCR, reversed transcriptase quantitative polymerase chain reaction; F, forward; R, reverse.*

### Functional Enrichment Analysis of Differentially Expressed Genes (DEGs)

For the function analysis, about 2,230, 1,689, and 1,812 DE mRNAs (*P* < 0.05, fold change ≥ 1.74), respectively, from the SC, ACC, and AMY were categorized using the Kyoto Encyclopedia of Genes and Genomes (KEGG pathway analysis) and Gene ontology analysis by the database for Annotation, Visualization and Integrated Discovery (DAVID^5^) (Li M. et al., 2020). Likewise, GO Annotations and KEGG Pathways Analysis were applied to predict the role of DE lncRNAs through their target mRNAs. The genetic regulatory networks were clarified by forming hierarchical categories according to the BP, MF, and CC aspects^6^ (Zhou et al., 2017). The significant pathway enrichments of differentially expressed lncRNAs were predicted by the Pathway Analysis^7^ (Zhou et al., 2017).

### Protein–Protein Interaction Network Construction

To further analyze and elucidate the functional connection between the differentially expressed encoding genes, the interaction among the significant DEGs in three regions was predicted by the STRING database (version: 11.0)^8^. The top 50 DEGs with the highest correlation degree were screened out to establish the network in the Cytoscape program (version: 3.6.0^9^) (Liu et al., 2019). The connection degree of each node was calculated through the cityscape plugin. The node size was defined by the connection degree. Red and blue colors represented the up- and downregulated genes, respectively.

### Co-expression Network Construction

LncRNA–mRNA co-expression networks were established based on the correlation between DE lncRNAs and their neighboring, overlapping, or distant mRNAs in the genome. DEGs with the Pearson correlation coefficients (PCC) > 0.95 or < –0.95 between lncRNAs and mRNAs (FDR < 0.05) were picked out to draw networks by the Cytoscape program (Dong et al., 2014).

### Statistical Analysis

All data were collected randomly and expressed as mean ± SEM. The data were statistically analyzed with two-tailed, unpaired Student’s *t*-test, and one-way and two-way ANOVA with repeated measures. When ANOVA showed a significant difference, pairwise comparisons between means were tested by the *post hoc* Tukey method. Values of *P* < 0.05 were considered statistically significant. The data were analyzed by GraphPad Prism 8.0.

## Results

### SNL Leads to Nociceptive Hypersensitivities and Pain Aversiveness Behaviors

**FIGURE 1:**
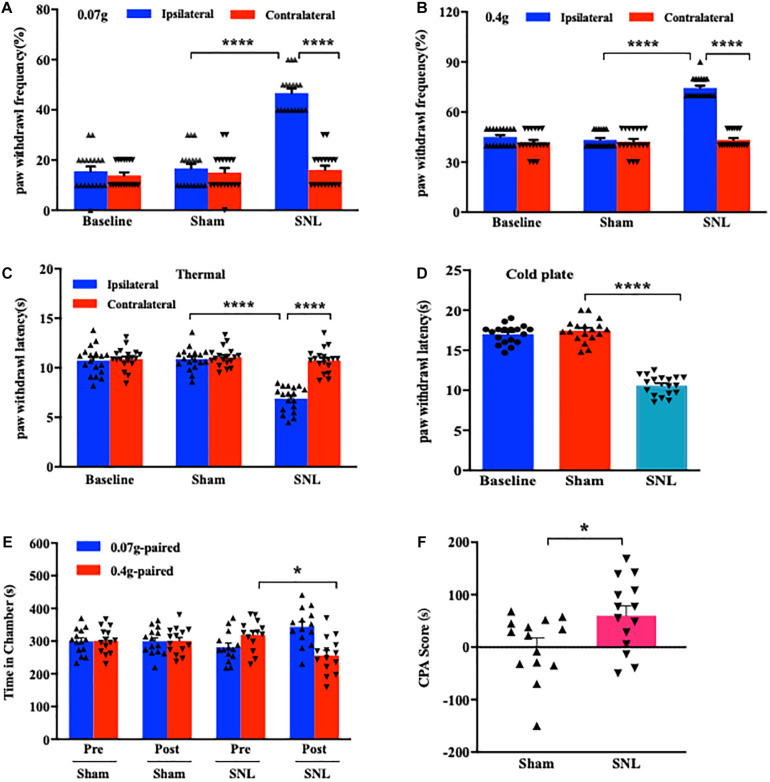
Unilateral L4 spinal nerve ligation (SNL) produced nociceptive hypersensitivities and pain aversiveness in mice. **(A,B)** Paw withdrawal frequencies in response to 0.07 g **(A)** and 0.4 g **(B)** von Frey filament stimuli on the ipsilateral and contralateral sides on day 7 following SNL or Sham surgery. *n* = 18/group. ****P < 0.0001 versus the corresponding sham group or the corresponding contralateral side by two-way ANOVA with repeated measures followed by *post hoc* Tukey test. **(C,D)** Paw withdrawal latencies in response to thermal **(C)** and cold **(D)** stimuli on the ipsilateral and contralateral sides on day 7 post-SNL or -Sham surgery. *n* = 18 mice/group for thermal test and 14 mice/group for the cold test. ****P < 0.0001 versus the corresponding sham group or the corresponding contralateral side by one-way **(D)** or two-way **(C)** ANOVA with repeated measures followed by *post hoc* Tukey test. **(E,F)** Time spent in the corresponding chamber paired with 0.07 g or 0.4 g von Frey filament stimuli **(E)** and CPA score **(F)** in the sham-operated and SNL-operated groups. *n* = 14 mice/group. Pre: Precondition. Post: Postcondition. N.S: not significant, *P* > 0.99. *P < 0.05 versus the corresponding precondition **(E)** or sham group **(F)** by two-way ANOVA with repeated measures followed by *post hoc* Tukey test **(E)** or two-tailed unpaired Student’s *t*-test **(F)**.

Consistent with early reports (Jiang et al., 2015; Wu et al., 2016), mice exposed to SNL showed significant mechanical allodynia (Figures 1A,B), thermal hyperalgesia (Figure 1C), and cold hyperalgesia (Figure 1D) as indicated by the increases in paw withdrawal frequencies in response to von Frey filament stimuli and the decreases in paw withdrawal latencies in response to heat and cold stimuli, respectively, on day 7 post-surgery on the ipsilateral side as compared to those on the contralateral side. As expected, no nociceptive hypersensitivities were observed on either side of sham-operated mice (Figures 1A–D). Previous studies indicated that peripheral nerve injury led to emotional aversion on day 3 postsurgery (Suzuki et al., 2007; Wu et al., 2019). In line with these studies (Guo et al., 2016; Wu et al., 2019), the time spent in the preferred chamber at initial was sharply reduced in SNL mice on day 7 postsurgery after the repeated stimuli of 0.4 g von Frey filament (Figure 1E; ^∗^*P* < 0.05), demonstrating that SNL mice exhibited emotional aversion. As expected, sham-operated animals did not show any differences when they received the same training (Figure 1E; *P* > 0.99). The difference in the CPA score between SNL- and sham-operated groups was statistically significant (Figure 1F). Taken together, SNL mice exhibited well-established nociceptive hypersensitivities and emotional aversiveness.

### RNA-Seq and Genome-Wide Read Mapping in the SC, ACC, and AMY After SNL

More than 50 million (M) reads in each group per region (SC: 55.88 M–95.96 M in sham and 80.78 M–86.10 M in SNL; ACC: 90.74 M-102.62 M in sham and 68.88 M–86.05 M in SNL; AMY: 98.44 M–111.45 M in sham; and 93.65 M–126.80 M in SNL) were achieved. After the trimmed reads were mapped to the reference mouse genome from ENSEMBLE (GRCm38.90), mapped reads were sorted through as exonic, intronic, and intergenic. The proportion of the reads within each category in sham and SNL groups from the SC, ACC, and AMY were illustrated in Supplementary Figure 1A. As expected, many reads were aligned to exonic regions in both groups followed by a considerable proportion of reads mapped to intronic regions (Supplementary Figure 1A). The reads mapped to intergenic regions accounted for a small percentage in both sham and SNL groups among the three regions (Supplementary Figure 1A). Furthermore, Supplementary Figures 1B,D illustrated a robust elevation in the level of reads mapped to the exonic region and a remarkable reduction in the proportion of reads aligning to intronic regions (^∗^*P* < 0.05; ^∗∗^*P* < 0.01) in the SC and AMY, implicating the changes in the functional proteins and signaling pathways after SNL. However, no significant changes were observed in the ACC (Supplementary Figure 1C).

We then analyzed the expression profiles of the DEGs in the SC, ACC, and AMY. Six days after SNL, approximately 38,584, 38,045, and 38,727 genes out of a total of 102,711, 101,218, and 102,589 transcripts, respectively, were identified in the SC, ACC, and AMY. The numbers of the changed genes in three regions were quite similar. In agreement with the changes in the injured DRGs (Wu et al., 2016), the largest transcriptional changes were observed in protein-coding RNAs (49%), followed by other non-coding RNAs (43–44%) and lncRNAs (7–8%) in three regions on day 7 after SNL (Supplementary Figures 1E–G).

### Altered Expression Profiles of mRNAs and lncRNAs in the SC, ACC, and AMY After SNL

**FIGURE 2:**
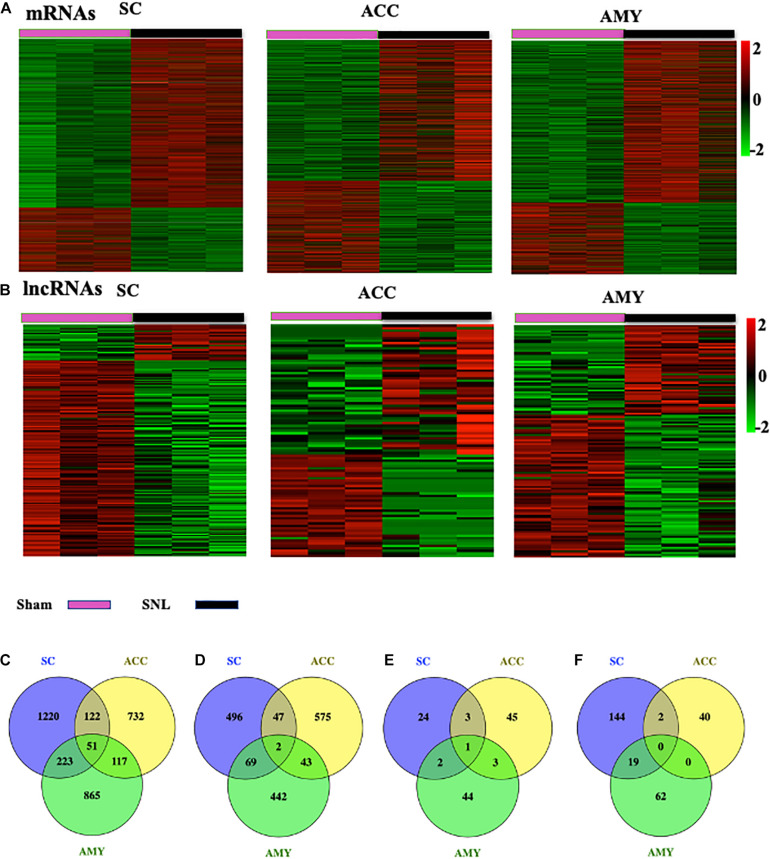
Differentially expressed mRNAs and lncRNAs in the SC, ACC, and AMY after nerve injury. **(A,B)** Heatmaps of significantly differentially expressed mRNAs **(A)** and lncRNAs **(B)** in the SC, ACC, and AMY from mice on day 7 post-SNL or -sham surgery. *n* = 9 mice/group. **(C,D)** Venn diagrams indicate the co-upregulated mRNAs **(C)** and co-downregulated mRNAs **(D)** in the SC, ACC, and AMY on day 7 postsurgery. *n* = 9 mice/group. **(E,F)** Venn diagrams indicate the co-upregulated lncRNAs **(E)** and co-downregulated lncRNAs **(F)** in the SC, ACC, and AMY on day 7 postsurgery. *n* = 9 mice/group.

The robust changes in gene expression of mRNAs and lncRNAs within the spinal cord, ACC, and AMY were observed after nerve injury. About 2,230 (1,616 upregulated, 614 downregulated), 1,689 (1,022 upregulated, 667 downregulated), and 1,812 (1,256 upregulated, 556 downregulated) mRNAs were significantly changed in the SC, ACC, and AMY, respectively (Supplementary Material 1). Besides, approximately 196 (30 upregulated, 136 downregulated), 94 (52 upregulated, 42 downregulated), and 131(50 upregulated, 86 downregulated) lncRNAs were significantly altered in the SC, ACC, and AMY, respectively (Supplementary Material 2). The clustered heatmaps of DE mRNAs (Figure 2A) and DE LncRNAs (Figure 2B) revealed distinct gene expression patterns across three regions after SNL.

Venn diagram was applied to further determine whether these DEGs showed co-expression patterns across three regions. The analyses characterized the co-expression genes by comparing up- and downregulated mRNAs and lncRNAs in the SC, ACC, and AMY. We found the co-regulation of DE mRNAs (Figures 2C,D) and DE lncRNAs (Figures 2E,F) in all three regions, but the robust co-expression patterns were seen in the co-upregulated mRNAs (Figure 2C), and the co-downregulated lncRNAs (Figure 2F), especially in the SC and AMY (Figures 2C,F). The detailed co-expressed DE mRNAs and DE lncRNAs were listed in Supplementary Materials 1, 2, respectively.

### Highest Differentially Expressed G Protein-Coupled Receptor mRNAs, Ion Channel mRNAs, and lncRNAs in the SC, ACC, and AMY After SNL

**FIGURE 3:**
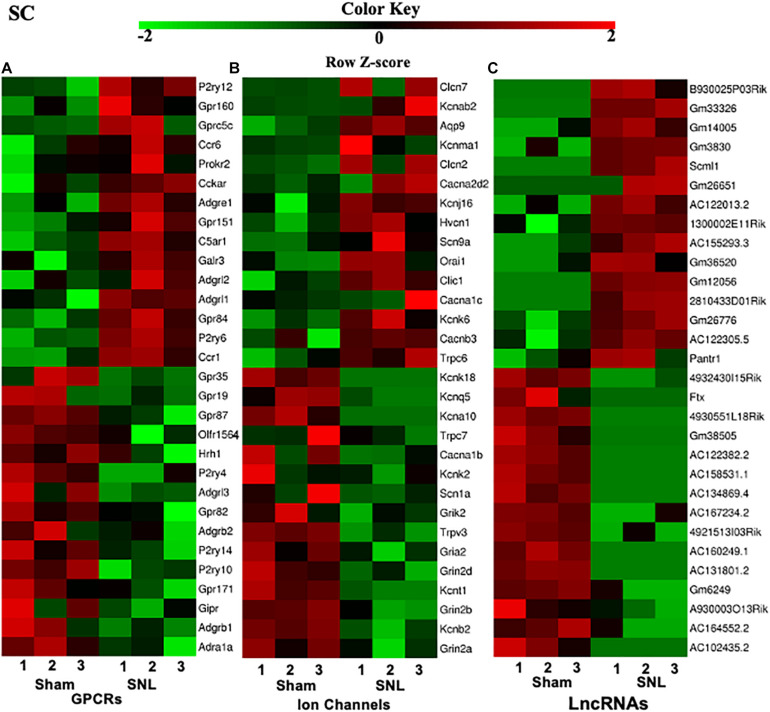
Heatmaps of the representative differentially expressed genes (DEGs) in the spinal cord after nerve injury. **(A-C)**, Top 15 up- and downregulated genes of G protein-coupled receptors **(A)**, ion channels **(B)**, and lncRNAs **(C)** in the fourth lumbar spinal cord on day 7 after SNL. Colors in the heatmaps indicate the Row Z-score among the different datasets. The up- and downregulated genes are colored in red and green, respectively. *n* = 9 mice/group.

G protein-coupled receptors (GPCRs), ion channels, and lncRNAs are critical in the transmission and modulation of nociceptive information (Zhao et al., 2013; Campbell and Smrcka, 2018; Klein and Oaklander, 2018). Besides 7–8% of the whole transcriptome that are lncRNAs, about 617, 552, and 598 DEGs were identified as GPCR mRNAs and 257, 253, and 250 DEGs were identified as ion channel mRNAs in the SC, ACC, and AMY, respectively. The top 15 up- and downregulated DEGs of GPCR, ion channel, and lncRNA across three regions were displayed in the heatmaps (Figure 3 and Supplementary Figures 2, 3). Consistent with previous reports, the levels of the GPCRs P2ry12 (Horváth et al., 2014), Gpr151 (Jiang et al., 2018), Prokr2 (Maftei et al., 2014), Ccr1 and Ccr5 (Eltayeb et al., 2007; Pevida et al., 2014) in the spinal cord (Figure 3A); D1 receptor (D1R) (Darvish-Ghane et al., 2020) and Cmklr1 (Guo et al., 2012; Doyle et al., 2014) in the ACC (Supplementary Figure 2A); and GPCRs C5ar2 (Carpanini et al., 2019) and Cckbr (Bowers and Ressler, 2015) in the AMY (Supplementary Figure 3A) were remarkably increased on day 7 after SNL. In contrast, the amounts of GPCR histamine receptor H1R (Huang et al., 2017) and G protein-coupled receptor 35 (Gpr35) (Cosi et al., 2011) in the spinal cord (Figure 3A), Grm5 (Ramos-Prats et al., 2019) in the ACC (Supplementary Figure 2A), and Gpr35 (Savitz et al., 2015) and Lpar1 (Pedraza et al., 2014; González de San Román et al., 2019) in the AMY (Supplementary Figure 3A) were dramatically decreased after SNL. For the ion channels, we observed the observably elevated expression of Cacna2d2 (Yu et al., 2019), Orai1 (Dou et al., 2018), Aqp9 (Wu et al., 2020), and Trpc6 (Jin et al., 2017; Wang et al., 2020) in the spinal cord (Figure 3A), Gria1 (Toyoda et al., 2009) and Cacna1c (Jeon et al., 2010) in the ACC (Supplementary Figure 2B), and Cacna1c (Temme and Murphy, 2017), Cacna2d1 (Chen et al., 2018; Young et al., 2016), and Trpc6 (Jin et al., 2017; Wang et al., 2020) in the AMY (Supplementary Figure 3B) after SNL. On the contrary, significant reductions were seen in the levels of ion channel transcripts for Kcnq5 (Manville and Abbott, 2018), Cacna1b (Stevens et al., 2019), and Kcnj6 (Zheng et al., 2015) in the spinal cord (Figure 3B), Gabrb3 (Tripp et al., 2012), Gabra1 (Chandley et al., 2015), and Grik2 (Chandley et al., 2015) in the ACC (Supplementary Figure 2B), as well as the downregulation of the ion channels such as Scn1a, Ano1, Cacna1h (Gangarossa et al., 2014), Cacna1d (McKinney et al., 2009), and Gabra1 (Guilloux et al., 2012) in the AMY (Supplementary Figure 3B) on day 7 after SNL. Interestingly, we detected the upregulation of P2ry12 mRNA in the AMY, which was inconsistent with an earlier report, in which the level of P2ry12 mRNA was unaltered in the AMY post-nerve injury (Barcelon et al., 2019).

Additionally, top 15 up- and downregulated lncRNAs in three regions were shown in heatmaps (Figure 3 and Supplementary Figures 2, 3C). As expected, some DEGs for lncRNAs that were identified in the present study (Table 2) have been previously reported to implicate in pain (Li et al., 2018; Tang et al., 2018; Che et al., 2019; Meng et al., 2019; Han et al., 2020; Wen et al., 2020; Wu et al., 2020). Consistently, we detected the increased expression of lncRNA Dancr and H19, and the decreased expression of lncRNA Meg3 in the spinal cord (Li et al., 2018; Tang et al., 2018; Che et al., 2019; Han et al., 2020; Wen et al., 2020). The downregulation of lncRNA Malat1 in the spinal cord and the upregulation of lncRNA Meg3 and lncRNA H19 in the ACC were observed on day 7 after SNL, but the functions of these lncRNAs under neuropathic pain conditions need to be further determined (Meng et al., 2019; Wu et al., 2020).

**TABLE 2:** Spinal nerve ligation-induced differentially expressed lncRNAs that have been previously reported implicated in pain.

Name	Description	Fold change	Ref.
Malat1	Metastasis-associated lung adenocarcinoma	0.68 (SC)	Meng et al., 2019; Wu et al., 2020
Meg3	Maternally expressed 3	0.22(SC); 2.14(ACC)	Li et al., 2018; Che et al., 2019
Dancr	Differentiation antagonizing non-coding RNA	6.34 (n.s) (SC)	Tang et al., 2018
H19	LncRNA H19	2.22(SC); 3.69(ACC)	Han et al., 2020; Wen et al., 2020

### Validation of the DEGs for lncRNAs and mRNAs in the SC, ACC, and AMY

**FIGURE 4:**
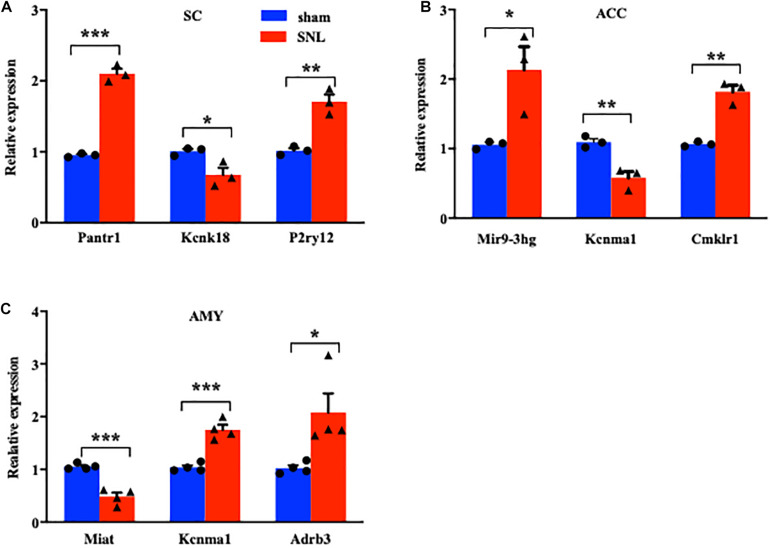
Validations of differentially expressed lncRNAs and mRNAs in the SC, ACC, and AMY after nerve injury. **(A)** Levels of lncRNA Pantr1, G protein-coupled receptor P2ry12, and ion channel Kcnk18 in the spinal cord on day 7 after SNL. *n* = 12 mice/group. *P < 0.05; **P < 0.01, and ***P < 0.001 versus the corresponding sham group by two-tailed unpaired Student’s *t*-test. **(B)** Amounts of lncRNA Mir9-3hg, G protein-coupled receptor Cmklr1, and ion channel Kcnma1 in the ACC on day 7 after SNL. *n* = 12 mice/group. *P < 0.05; **P < 0.01, versus the corresponding sham group by two-tailed unpaired Student’s *t*-test. **(C)** Levels of lncRNA Miat, G protein-coupled receptor Adrb3, and ion channel Kcnma1 in the AMY on day 7 after SNL. *n* = 12 mice/group. *P < 0.05; **P < 0.01, and ***P < 0.001 versus the corresponding sham group by two-tailed unpaired Student’s *t*-test.

We next conducted a quantitative real-time RT-PCR assay to validate the reliability of RNA sequencing results by analyzing the expression of significant DE lncRNAs and mRNAs on day 7 after SNL in three regions. The expression of three lncRNAs (Pantr1, Mir9-3hg, and Miat), two ion channel mRNAs (Kcnk18 and Kcnma1), and three G protein-coupled receptor mRNAs (P2ry12, Cmklr1, and Adrb3) was measured in the SC (Figure 4A), ACC (Figure 4B), and AMY (Figure 4C), respectively. As expected, the levels of the selected lncRNAs and mRNAs were concomitant with the sequencing results (Figures 4A–C). It was noteworthy that the amount of Kcnma1 was elevated in the AMY (Figure 4C) but reduced in the ACC (Figure 4B).

### Functional Enrichment Analysis of the Differentially Expressed Genes After SNL

**FIGURE 5:**
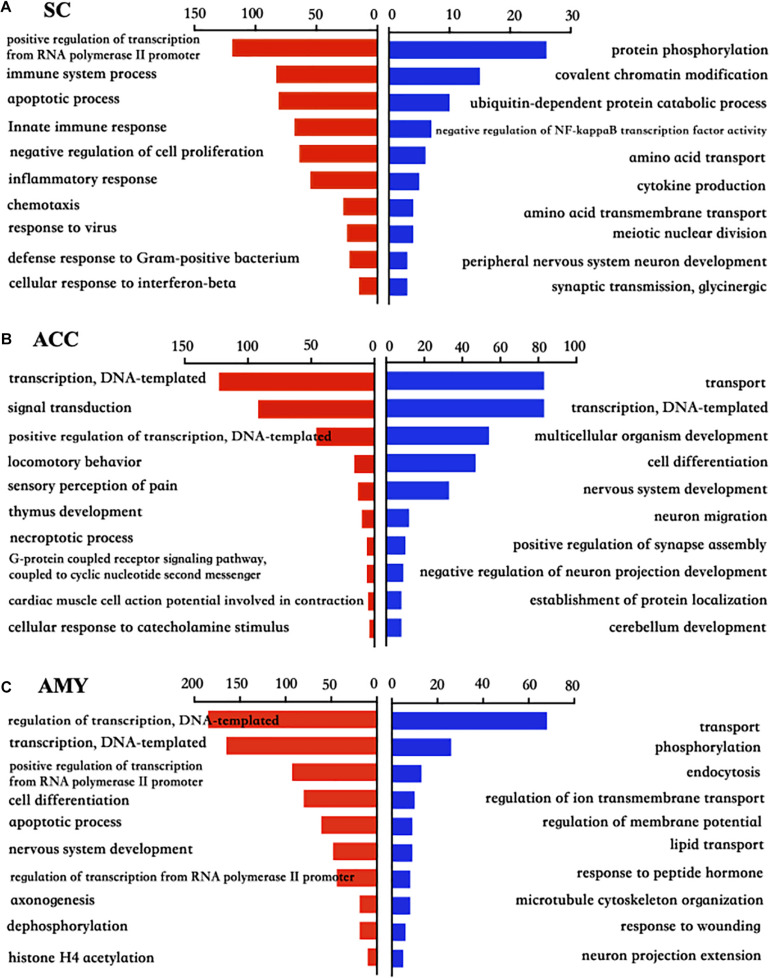
Biological process analysis of the differentially expressed up- and downregulated mRNAs in the SC, ACC, and AMY after nerve injury. **(A–C)** Analysis of the Gene Ontology database showed top 10 biological processes from upregulated mRNAs (red panels on the left) and downregulated mRNAs (blue panels on the right) in the SC **(A)**, ACC **(B)**, and AMY **(C)** on day 7 post-SNL according to the *P*-value. The DAVID database was used to do the GO enrichment analysis. Red and blue bars represent up- and downregulated mRNA enrichments, respectively.

To explore the functional enrichments of these DEGs, we performed Gene Ontology and KEGG pathway analyses to categorize the up- and downregulated mRNAs based on the distinct processes using the DAVID bioinformatics database. The top 10 analyzed results of biological processes from the up- (red panels on the left) and downregulated (blue panels on the right) mRNAs in three regions were displayed in Figure 5 and Supplementary Material 3. The most significant enriched biological processes of upregulated genes in the spinal cord were immune system process, apoptotic process, innate immune process, inflammatory response, defense response, regulation of RNA transcription, and cell proliferation, while the downregulated genes in the spinal cord were mainly involved in protein phosphorylation, covalent chromatin modification, negative regulation of NF-kappaB transcription factor activity, and cytokine production after nerve injury (Figure 5A). The upregulated DEGs in the ACC were highly enriched in transcription, regulation of transcription, signal transduction, locomotory behavior, and sensory perception of pain and G protein-coupled receptor pathways, in contrast to the prominent enrichments in transport, cell differentiation, neuron migration, and positive regulation of synapse assembly for downregulated genes (Figure 5B). The upregulated genes in the AMY were related to cell proliferation, apoptotic process, nerve system development, and Histone H4 acetylation besides the enrichments in transcription-related processes, whereas the downregulated genes in the AMY were markedly enriched in regulation of membrane potential, response to wounding, and neuron projection extensive as well as transport processes (Figure 5C). For the molecular function enrichments, we observed the striking enrichments in protein binding, DNA binding, and protein homodimerization activity for the upregulated genes and metal ion binding, ATP binding, and transferase activity for downregulated genes in the spinal cord (Supplementary Material 3). In the ACC, the upregulated genes were prominently implicated in protein binding, action binding, ion channel binding, and protein kinase binding, while the downregulated genes were involved in protein binding, transcription factor binding, and DNA binding (Supplementary Material 3). In the AMY, the upregulated genes were distinctly enriched in protein binding, protein N-terminal binding, and chromatin binding, while the downregulated genes were mainly enriched in lipid binding, endopeptidase inhibitor activity, and protein homodimerization activity (Supplementary Material 3). Within the category of “cellular component,” the DEGs in three regions were robustly enriched in membrane, cytoplasm, and nucleus (Supplementary Material 3).

**FIGURE 6:**
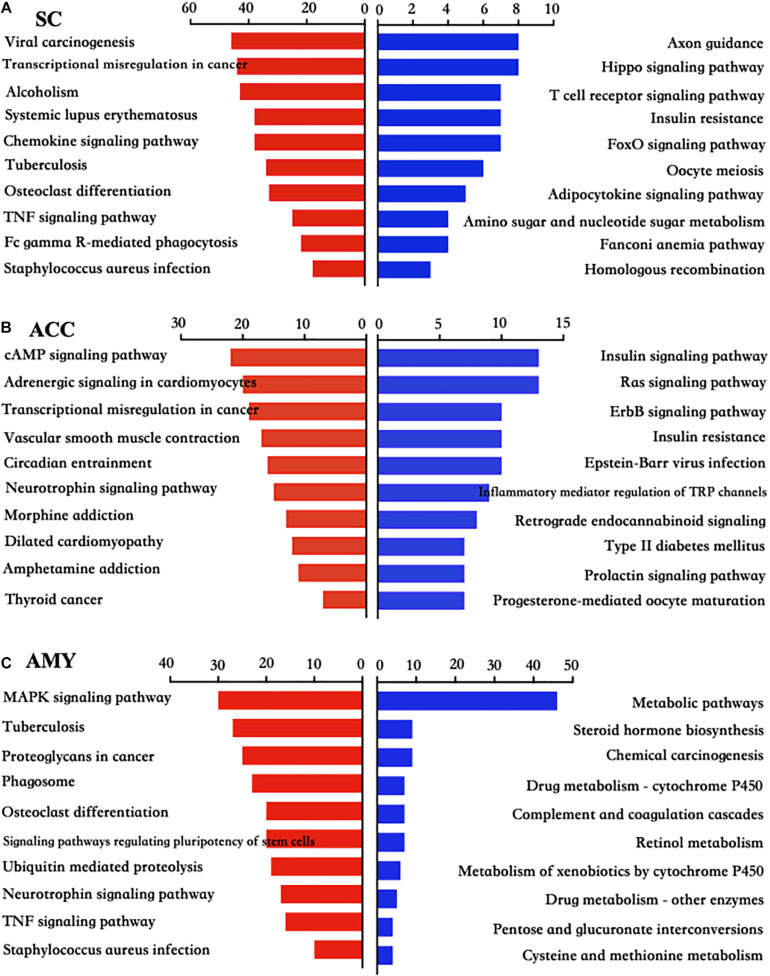
KEGG pathway analysis of the differentially up- and downregulated mRNAs in the SC, ACC, and AMY after nerve injury. **(A–C)** The top 10 enrichments of KEGG pathways from upregulated mRNAs (red panels on the left) and downregulated mRNAs (blue panels on the right) in the SC **(A)**, ACC **(B)**, and AMY **(C)** on day 7 post-SNL according to the *P*-value. The DAVID database was used to do the KEGG pathway analysis. Red and blue bars represent up- and downregulated mRNA enrichments, respectively.

Pathway analyses showed that most significant pathway enrichments in the spinal cord contained chemokine signaling pathway, tumor necrosis factor (TNF) signaling pathway, and Fc gamma R-mediated phagocytosis for the upregulated genes (red panels on the left) and axon guidance, hippo signaling pathway, and T cell receptor signaling pathway for the downregulated genes (blue panels on the right) (Figure 6A). In the ACC, the obvious enrichments were seen in the cAMP signaling pathway, neurotrophin signaling pathway and morphine addiction for the upregulated genes and the insulin signaling pathway, Ras signaling pathway, inflammatory mediator regulation of TRP channels, and type II diabetes mellitus for the downregulated genes (Figure 6B). In the AMY, the dramatic enrichments were detected in the MAPK signaling pathway, osteoclast differentiation, TNF signaling pathway for the upregulated genes and the metabolic pathways, chemical carcinogenesis, and complement and coagulation cascades for the downregulated genes (Figure 6C). These findings indicated the overlapped function in biological processes and pathways among three regions, which was further verified by the more detailed function analyses by comparing the function enrichments of the overall DE mRNAs in three regions (Supplementary Figure 4).

### PPI Network Establishment to Analyze Protein–Protein Interactions in Three Regions After SNL

**FIGURE 7:**
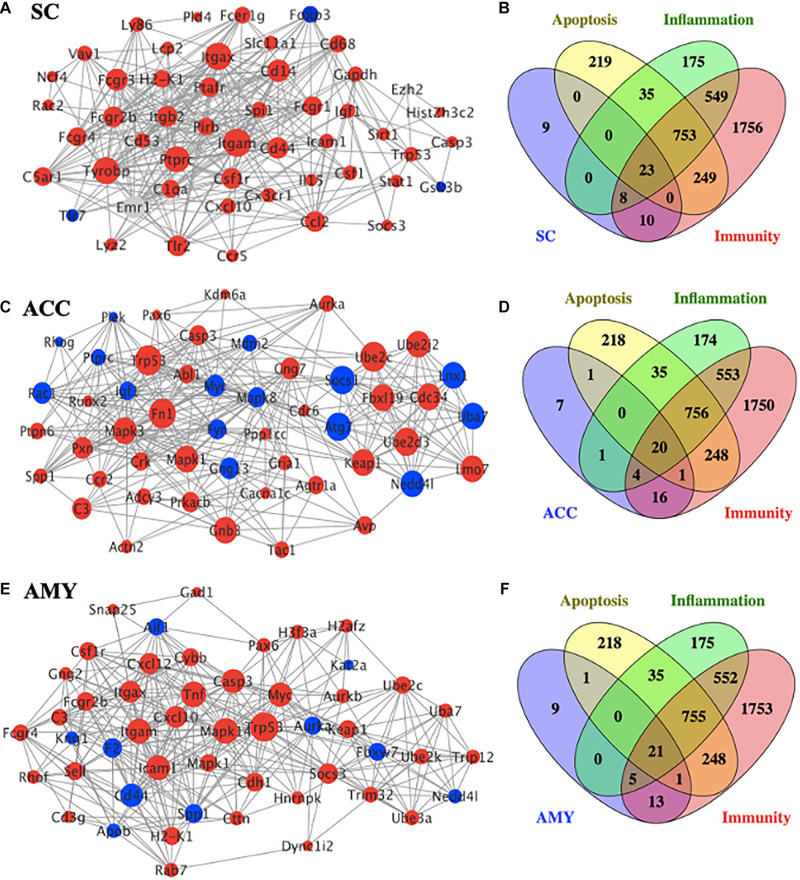
PPI network establishments to analyze protein–protein interactions. (**A**,**C**,**E**) Top 50 differentially expressed genes (DEGs) were picked out based on the connection degree of genes and constructed the network in the SC **(A)**, ACC **(C)**, and AMY **(E)**. The size of the node represents the connection degree that indicates the importance of the gene in the network. Red and blue colors represent the up- and downregulated genes, respectively. (**B**,**D**,**F**) Venn diagrams indicated the number and proportion of the selected top 50 DEGs mapped to apoptosis-, inflammation-, and immunity-related genes in the SC **(B)**, ACC **(D)**, and AMY **(E)**, respectively.

To gain insight into the functional connection among the DE mRNAs and their potential role in neuropathic pain, a PPI network was conducted using the STRING database. The top 50 protein-coding DEGs with the highest correlation degree in each region were screened out and used to generate the network. As shown in Figure 7A, the increased DEGs, such as Itgam, Itgax, Tyrobp, Ptprc, Cd14, Fcgr3, and Cd44, were the crucial molecules among the hub genes in the network of SC, whereas the increased DEGs, such as Trp53, Mapk1, Mapk3, Fn1, Cnb3, Ube2c, Ube2d3, Fbxl19, Cdc34, Keap1, and Lmo7, and the decreased DEGs, such as Atg7, Socs1, Lnx1, Nedd4l, and Uba7, played a major role in the network of ACC (Figure 7C). The hub genes in the network of AMY (Figure 7E) revealed the vital position of Trp53, Mapk14, Tnf, Icam1, Itgan, Casp3, Myc, and Cd44. In addition, considering that neuroinflammation and apoptosis participated in many pathological processes including neurological and psychiatric disorders (Chelyshev et al., 2001; Ji et al., 2018; Matsuda et al., 2019), we defined the function of top 50 DEGs in the network of SC, ACC, and AMY by comparing them with a total number of 3,773 genes related to neuroinflammation (inflammation and immunity) and apoptosis (1,543, 3,348, and 1,279 related genes for inflammation, apoptosis, and immunity, respectively). Venn diagrams showed that approximately 82%, 86%, and 82% of DEGs, respectively, from the SC, ACC, and AMY were mapped to neuroinflammation- and apoptosis-related genes (Figures 7B,D,F).

### Differentially Expressed mRNAs Implicated in Pain, Anxiety, and Depression Disorders Across Three Regions After SNL

**FIGURE 8:**
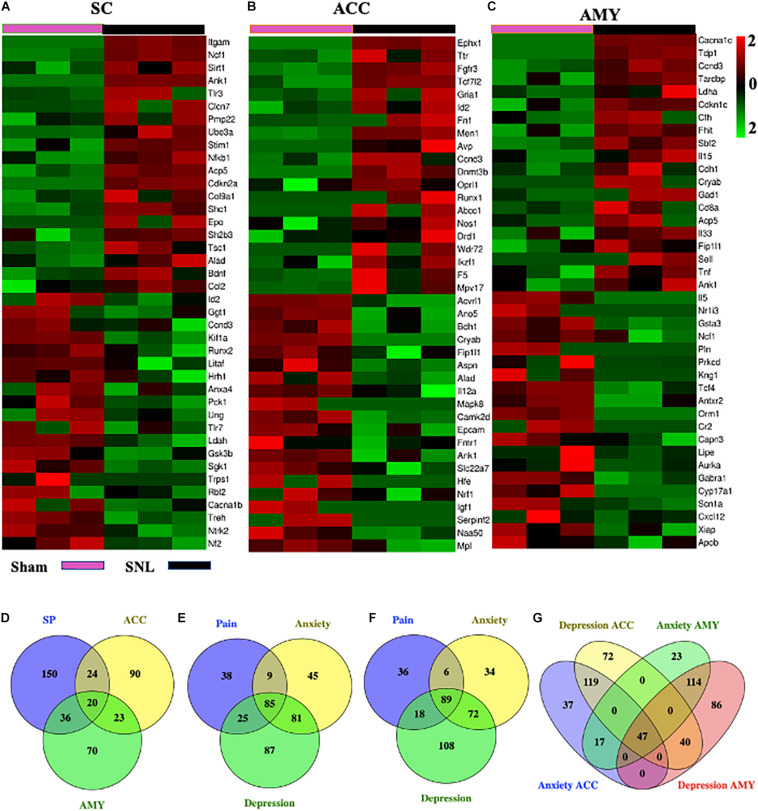
Comparisons of pain-, anxiety-, and depression-related genes in the SC, ACC, and AMY. **(A–C)** Heatmaps of representative top 20 up- and downregulated pain-related DEGs in the SC **(A)**, ACC **(B)**, and AMY **(C)** on day 7 after SNL. **(D)** Venn diagram indicated the number of the overlapped pain-related genes among the SC, ACC, and AMY on day 7 after SNL. Detailed information was displayed in Supplementary Table 3. **(E–G)** Venn diagrams indicated the number of overlapped pain-, anxiety-, and depression-related DEGs in the ACC and AMY (detailed information in Supplementary Table 4). Colors in the heatmaps indicate the Row Z-score among the different datasets. Up- and downregulated genes are colored in red and green, respectively.

We next used the Gene Cards database and CTD database to characterize the DEGs involved in pain and emotional disorders in the SC, ACC, and AMY. Based on the relevance score, about 230 (196 upregulated, 34 downregulated), 157 (100 upregulated, 57 downregulated), and 149 (100 upregulated, 49 downregulated) pain-related DEGs in the SC, ACC, and AMY, respectively, were observed (Supplementary Figure 5 and Supplementary Material 4). The ACC and AMY contained approximately 220 (140 upregulated, 80 downregulated) and 201 (146 upregulated, 55 downregulated) anxiety-related DEGs as well as about 278 (176 upregulated, 102 downregulated) and 287 (207 upregulated, 80 downregulated) depression-related DEGs, respectively (Supplementary Figure 5 and Supplementary Material 5). The top 20 highest up- and downregulated pain-related DEGs were displayed in heatmaps (Figures 8A–C).

### Pain-, Anxiety-, and Depression-Related DEGs Displayed a High Correlation to Neuroinflammation and Apoptosis in Three Regions After SNL

To further determine the function of DEGs in SNL-induced neuropathic pain pathogenesis, we investigated the correlation between the DEGs implicated in pain, depression, and anxiety disorders and the genes related to neuroinflammation (inflammation and immunity) and apoptosis. About 230 pain-related genes in the SC, 157 pain-related genes, 220 anxiety-related genes, and 278 depression-related genes in the ACC and 149 pain-related genes, 201 anxiety-related genes, and 287 depression-related genes in the AMY were mapped to the neuroinflammation- (inflammation and immunity) and apoptosis-related genes. There were about 51%, 60%, and 71% of pain-related DEGs in the SC; 42%, 52%, and 58% of pain-related DEGs in the ACC; and 47%, 59%, and 68% of pain-related DEGs in the AMY mapping to the datasets of apoptosis, inflammation, and immunity, respectively (Supplementary Table 1). Approximately 179, 99, and 110 pain-related overlapping DEGs were seen in three distinct regions, respectively (Supplementary Table 1). About 26%, 30%, and 36% of anxiety-related DEGs in the ACC; 31%, 36%, and 49% of anxiety-related DEGs in the AMY; 28%, 30%, and 41% of depression-related DEGs in the ACC; and 27%, 30%, and 44% of depression-related DEGs in the AMY were mapped to the genes associated with apoptosis, inflammation, and immunity, respectively (Supplementary Table 2). Furthermore, we found about 88 and 106 overlapping anxiety-related DEGs and 125 and 139 overlapping depression-related DEGs in the ACC and AMY, respectively (Supplementary Table 2).

### Comparisons of Pain-, Anxiety-, and Depression-Related DEGs Among the SC, ACC, and AMY After SNL

To obtain more information about gene adaptations in response to SNL, we compared pain-related DEGs among three distinct regions in the present study (Figure 8D). Strikingly, about 103 overlapping genes were seen among the SC, ACC, and AMY (Supplementary Table 3). Of them, we observed the increased expression of C3, Cacna1c, Casp3, Cfh, Crem, Fn1, Men1, Oprl1, Sparc, Trp53, and Txnip and the decreased expression of Scn1a in all three regions. Interestingly, Scn1a was also reported downregulated in injured DRGs (Wu et al., 2016). In addition to the concordant expression patterns, we saw the discrepancy in the expression of the genes such as Alad, Ank1, Aurka, Ccnd3, Hgf, Mtm1, Nrf1, Tcf4, Wnk1, and Tardbp among the SC, ACC, and AMY. Consistent with the earlier findings in the injured DRGs (Wu et al., 2016), the expression of Gabra1 was reduced and the expression of Cacna2d1 was elevated, both in ACC and AMY after SNL. We also detected the upregulation of Acadvl, Acp5, Aifm1, Crh, Crlf1, Cxcl10, Deaf1, Eif4g1, Elane, F13a, Fgfr1, Fgfr3, Fhit, Gnas, Icam1, Il15, Il31ra, Itgam, Lgals1, Socs3, Stim1, Trappc2, Tsc22d3, Ube3a, Vgf, Vim, and Vip and the downregulation of Kif1a and Lipe in both SC and AMY. Finally, there were also many overlapped genes between the SC and ACC including the upregulated expression of Ikzf1, Pdyn, Plaur, Ppp1r1b, Pygl, and Sh2b3 and the downregulated expression of Anxa4, Cacna1b, Hrh1, Nf2, and Sgk1, as well as the inconsistent expression patterns in Arnt, Capn3, Ccr6, Cnbp, Igf1, Mapk8, Nfkb1, Id2, Litaf, Runx2, Tpm1, and Trps1.

Moreover, pain-, anxiety-, and depression-related DEGs in the ACC and AMY were compared (Figures 8E–G), A total of 338 pain-, anxiety-, and depression-related DEGs were observed in the ACC and AMY (Supplementary Table 4). Besides pain-related DEGs mentioned above, we saw the upregulated expression of Camk2b, Ccnd3, Ccng2, Cyp26b1, Fkbp5, Fgfr3, Gcnt2, Gnas, Gria1, Keap1, Map2, Mapk1, Mapk9, Nr4a2, Nr4a3, Oprk1, Oprl1, Pde4b, Pde7b, Ppfibp1, Psrc1, Tpm1, Tpm3, and Ube2c mRNAs and the downregulated expression of Tcf4, Pln, and Mtm1 mRNAs in both ACC and AMY. Additionally, the genes such as Cryab, Dclk1, Eif5a, Hgf, Magi1, Mef2c, Myc, Nr3c1, Plec Rai1, and Tardbp were downregulated in the ACC but upregulated in the AMY. The genes including Arrdc3, Diablo, Fmo5, Fnbp1, Ggt1, Nos1, Postn, Tcf7l2, and Xiap were significantly elevated in the ACC but reduced in the AMY. These findings suggested some shared pathogenesis mechanisms in neuropathic pain and emotional disorders, probably providing more evidence and basis for future researches.

### Functional Prediction of DE lncRNAs in SNL

We next determined the function of lncRNAs through their related mRNAs by selecting the genes with the absolute value of correlation > 0.95 and the co-localization within 100 kb at the upstream and downstream (Supplementary Material 6).

**FIGURE 9:**
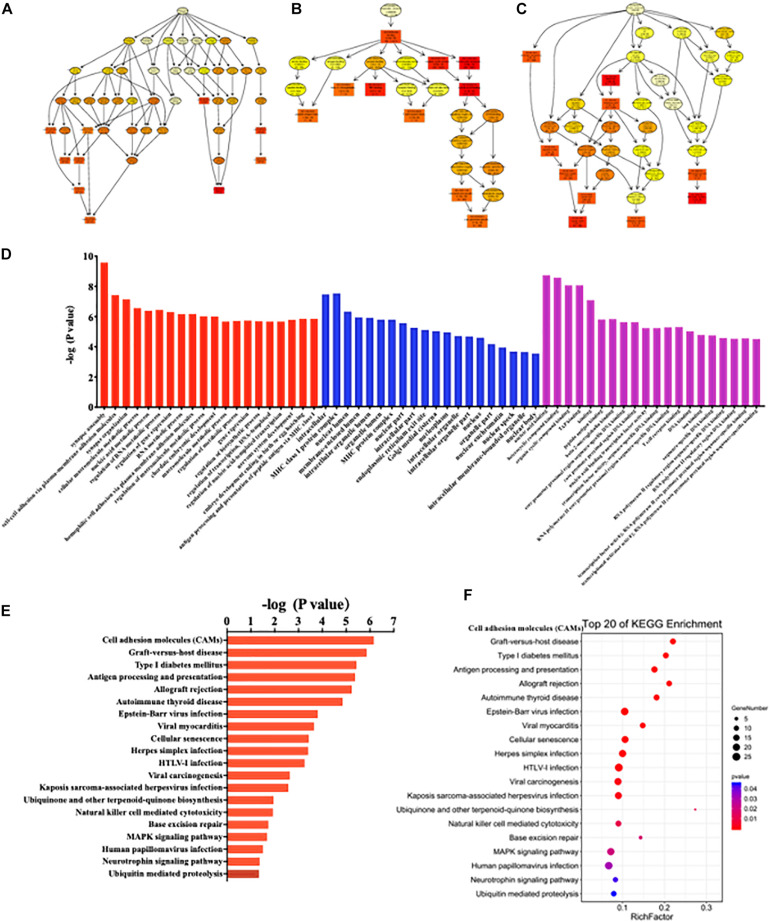
Functional prediction of DE lncRNAs by GO and KEGG analyses in the spinal cord of SNL mice. **(A–C)** Directed acyclic graphs (DAGs) graphically display the significant GO enrichment results with the candidate targeted genes in biological process **(A)**, molecular function **(B)**, and cellular component **(C)**. **(D)** Significant molecular function, biological process, and cellular component enrichment analysis of DE lncRNA-related mRNAs. The enrichment scores (−log10 (*P*-value)) of the GO term were shown in the histogram. **(E,F)** The DE lncRNA-related mRNA-enriched KEGG pathways represented by enrichment scores (−log10 (*P*-value)) **(E)** and the scatterplot showing statistics of pathway enrichment **(F)**, respectively. The color of pathway terms is defined by the enrichment *P*-value.

GO enrichment analysis results were graphically displayed in directed acyclic graphs (DAGs), in which the branch represented the relationship of the inclusion that defined the smaller and smaller scales from top to bottom. The top 10 GO enrichments were selected as the master nodes of DAGs. They were shown together with the GO terms of containment relationships and systematically GO terms. DAGs were plotted from the biological process, molecular function, and cellular component aspects in the SC, ACC, and AMY, respectively (Figure 9 and Supplementary Figures 6, 7A-C). According to the distribution of predicted target genes in the Gene Ontology, the function of DE lncRNAs was clarified and displayed in the form of histograms by the -log (*P*-value) of each GO term. Apparently, the most significant biological process enrichments were synapse assembly, cell–cell adhesion via plasma membrane adhesion molecules, synapse organization, cellular macromolecule metabolic process, nucleic acid metabolic process, and regulation of RNA metabolic process in three regions. The noteworthy cellular component enrichments were seen in intracellular, MHC class I protein complex, nuclear lumen, membrane-enclosed lumen, and intracellular organelle lumen in the SC, ACC, and AMY. The most robust molecular functions were enriched in binding, nucleic acid binding, heterocyclic compound binding, organic cyclic compound binding, and TAP binding among three regions (Figure 9 and Supplementary Figures 6, 7D).

Similarly, the top 20 KEGG enrichments were shown in the histograms by the -log (*P*-value) of each pathway (Figure 9 and Supplementary Figures 6, 7E) and together with the enriched distribution maps (Figure 9 and Supplementary Figures 6, 7F), in which the degree of KEGG enrichment was assessed by the Rich factor, *P*-value, and the number of genes. The enrichment was more significant with the greater rich factor and the larger number of genes but the smaller *P*-value. The most significantly enriched pathways were related to cell adhesion molecules (CAMs), graft-versus-host disease, type 1 diabetes mellitus, antigen processing and presentation, allograft rejection autoimmune thyroid disease, and cellular senescence among three regions after SNL (Figure 9 and Supplementary Figures 6, 7F). Overall, these data demonstrated the overlapped effects in DE lncRNA function among three regions, similar to the patterns of the DE mRNAs.

### lncRNA–mRNA Co-expression Network Analysis

**FIGURE 10:**
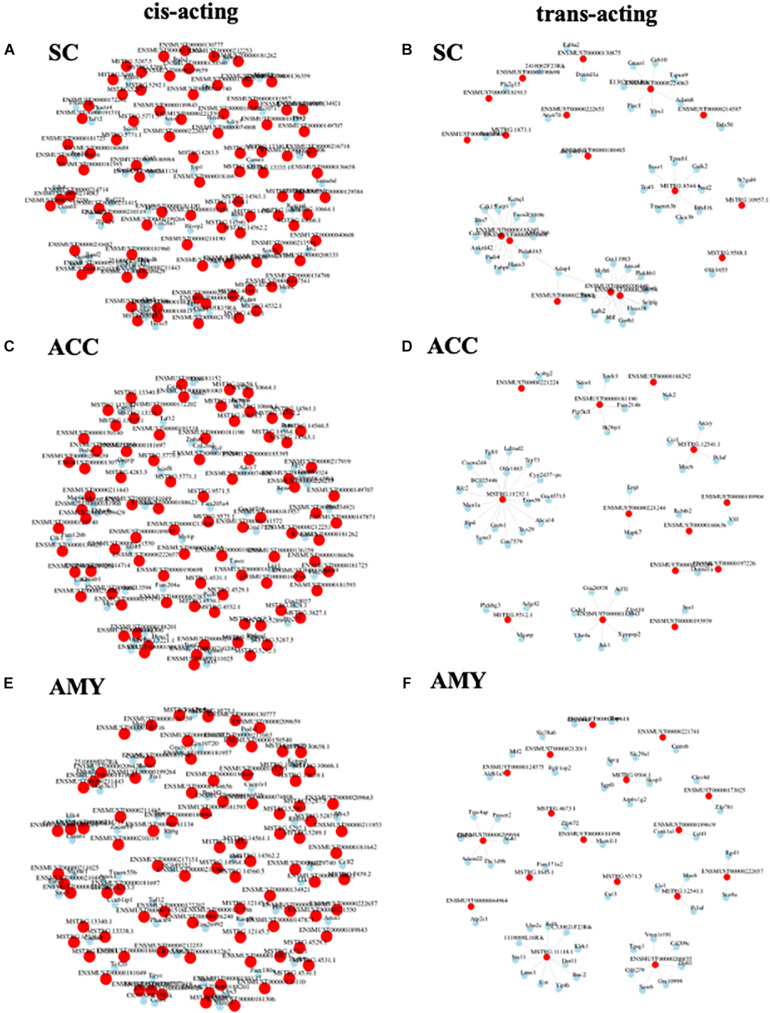
Analysis of lncRNA–mRNA co-expression network in the SC, ACC, and AMY of SNL mice. **(A,B)** Co-expression network of cis-acting regulatory elements **(A)** and trans-acting regulatory elements **(B)** in the SC. **(C,D)** Co-expression network of cis-acting regulatory elements **(C)** and trans-acting regulatory elements **(D)** in the ACC. **(E,F)** The co-expression network of cis-acting regulatory elements **(E)** and trans-acting regulatory elements **(F)** in the AMY. LncRNA–mRNA co-expression network was constructed based on the Pearson correlation coefficients. Pink color nodes represent lncRNA, while blue nodes represent the co-expression mRNAs.

To observe the potential interaction between lncRNAs and mRNAs in three regions after SNL, gene co-expression networks were constructed based on the correlation analysis. The networks were established by numbers of DE lncRNAs and the most potential top 50 DE mRNAs targets (PCC > 0.95 or <–0.95, and *FDR* < 0.05) (Supplementary Material 7). The cis-acting regulatory networks were constructed with 136 relationships between 88 lncRNAs (61 known, 26 predicted) and top 50 mRNAs in the SC (Figure 10A), 137 relationships between 87 lncRNAs (56 known, 31 predicted) and top 50 mRNAs in the ACC (Figure 10C), and 137 relationships between 89 lncRNAs (59 known, 30 predicted) and top 50 mRNAs in the AMY (Figure 10E). In contrast, the co-expression networks for trans-acting regulation consisted of 80 relationships between 17 lncRNAs (13 known, 4 predicted) and top 50 mRNAs in the SC (Figure 10B), 45 relationships between 13 lncRNAs (10 known, 3 predicted) and top 50 mRNAs in the ACC (Figure 10D), and 51 relationships between 17 lncRNAs (11 known, 6 predicted) and top 50 mRNAs in the AMY (Figure 10F).

## Discussion

Neuropathic pain is a somatosensory disorder resulting from nerve injury or diseases affecting the peripheral and central nervous systems (Colloca et al., 2017). With the high incidence and poor management in the clinic, it is a major public health problem. Over the past decades, the potential mechanisms underlying neuropathic pain have been extensively studied. However, the effective treatments are still limited due to the largely unknown molecular mechanisms (Finnerup et al., 2015; Jones et al., 2018). Evidence demonstrates that the alteration in gene expression profiles at different levels of the nervous system plays an important role in the development and maintenance of neuropathic pain. In the present study, we reported gene transcript alterations in pain- and emotion-associated regions in the central nervous system following peripheral nerve injury in mice using the next-generation RNA sequencing assay. Bioinformatics and pathway analyses revealed that particular differentially expressed gene patterns and biological networks in the SC, ACC, and AMY were in correspondence with SNL-induced nociceptive hypersensitivities and pain-related aversion.

In the present study, numerous DEGs belonging to GPCR and ion channel genes were identified. Many of these DEGs have identified function in pain or emotion dysfunction. Ccr1 and Ccr5 were shown to be involved in heat hyperalgesia in mice (Eltayeb et al., 2007; Pevida et al., 2014). The modification of Cmklr1 was shown to implicate depression in the prefrontal cortex and hippocampus responding to chronic restraint stress (CRS) (Guo et al., 2012; Doyle et al., 2014). Genetic knockout of Orai1 nearly eliminated the second phase of formalin-induced pain and attenuated carrageenan-induced pain hypersensitivity and neuronal excitability (Dou et al., 2018). TRPC6 inhibition in the spinal cord blocked the induction of morphine tolerance and hyperalgesia in rats (Jin et al., 2017; Wang et al., 2020). Likewise, we identified the changes in lncRNA expression after SNL. The expression of lncRNA Malat1 was controversial. Following our sequencing result, Meng C et al. demonstrated that the inhibition of spinal Malat1 expression contributed to neuropathic pain after brachial plexus avulsion (Meng et al., 2019). On the contrary, another study reported that the inhibition of spinal Malat1 reduced the incidence of CCI-induced neuropathic pain (Wu et al., 2020). These findings suggest that the molecular mechanism underlying neuropathic pain may vary with different etiologies and courses. LncRNA H19 was upregulated in the injured DRGs and hippocampus neurons following peripheral nerve injury (Han et al., 2020; Wen et al., 2020). Consistently, our sequencing data showed that SNL increased its expression in the spinal cord and ACC. However, whether the increased H19 in these two regions contributes to neuropathic pain needs to be confirmed.

Furthermore, lots of pain-, anxiety-, and depression-related genes were identified in the SC, ACC, and AMY following SNL. Consistent with previous studies, Itgam (CD11b) (Ghasemlou et al., 2015), Tlr3 (Liu et al., 2012), Bdnf (Sapio et al., 2019), and Stim1 (Gao et al., 2016) were significantly elevated, while Kif1a (Wang et al., 2018) and Rbl2 (Chen et al., 2019) were robustly decreased in the spinal cord. However, there was a discrepancy in the expression of Gsk3b. As reported, Gsk3b was downregulated at day 3 and upregulated at day 10 in the spinal cord after partial sciatic nerve ligation (Weng et al., 2014). Unexpectedly, we saw the decreased expression of spinal Gsk3b on day 7 after SNL. These results may imply that spinal Gsk3b expression is time-dependent post-nerve injury. In the ACC and AMY, we observed the upregulation of Drd1 (Darvish-Ghane et al., 2020), Tnf (Akter et al., 2020), and Il33 (Fairlie-Clarke et al., 2018), as well as the increased expression of Runx1 and Cd68 among pain-related DEGs. However, the function of Runx1 and Cd68 in these two regions is still unknown and remains to be investigated.

According to the sequencing data from the injured DRGs (Wu et al., 2016), we observed similar expression changes of some genes such as the upregulation of Atf3, Ccr1, Ccr5, and Gal, and the downregulation of Gria2 in the spinal cord as well as the elevated expression of Cacna2d1 and the reduced expression of Gabra1 in the ACC and AMY after SNL. Besides, we found the consistent expression patterns of some genes such as the upregulation of Atf3, Bdnf, C1qa, C3, Cck, Ccl2, Cd68, Csf1, Cx3cr1, Gch1, Itgam, Ngfr, Pdyn, and Tlr2 in the spinal cord of SNL mice, when compared to the sequencing results in CCI rats (Du et al., 2018; Korczeniewska et al., 2020). These data suggest the common gene expression patterns independent of regions, models, or species. The ACC and AMY are important brain areas in pain and emotion modulation, but the gene expression profiles in these two regions after nerve injury have not been identified before. We achieved more anxiety- and depression-related DEGs than pain-related DEGs in these two regions, consistent with their significant function in emotion processing. Interestingly, among the anxiety- and depression-related DEGs, we found the upregulation of Bcl3, C3, Gpat3, and Tnf in the AMY as well as the increased expression of Crhr2, Nant, Sar1a, and Tgif1 and the decreased expression of Ier2, Il12a, Nrep, and Tnfrsf25 in the ACC. These alternations were in step with the sequencing results in 12- and 24-month-old mice (Li M. et al., 2020), suggesting some common gene expression alterations in different pathological processes, at least for anxiety- and depression-related genes.

Consistent with previous reports (Jiang et al., 2015; Wu et al., 2016), GO term and KEGG pathway enrichment analyses in three regions showed notable enrichments in apoptotic, inflammation, immunity, cytokine production and defense response, behavior, and sensory perception of pain as well as the enrichments in chemokine signaling pathway, MAPK signaling pathway, TNF signaling pathway, cAMP signaling pathway, Type II diabetes mellitus, and T cell receptor signaling pathway. Consistently, functional analyses observed that large percentages of pain-, anxiety-, and depression-related DEGs were highly related to neuroinflammation and apoptosis that were considered to occupy an important position in pain states (Chelyshev et al., 2001; Ji et al., 2018; Matsuda et al., 2019). Among the overlapped pain-related DEGs, the amounts of Cacna1c, Casp3, C3, and TXNIP were sharply elevated in all three regions following SNL. ACC-conditional deletion of Cav1.2 channels impaired observational fear learning and reduced behavioral pain responses, while neuronal deletion of Cav1.2 led to significant deficits in the extinction of conditioned fear and altered sIPSC and sEPSC activity within the amygdala (Jeon et al., 2010; Temme and Murphy, 2017). Evidence indicated that Casp3 was upregulated in neuropathic pain and that the activation of Casp3 was required in long-term depression (Li et al., 2010; Yang et al., 2018). The deletion of complement C3 was shown to reduce pain-, anxiety-, and depression-like behaviors and to improve learning and spatial memory in aged mice (Shi et al., 2015; Crider et al., 2018). Recent reports suggested that activation of TXNIP/NLRP3 axis was positively associated with pain and emotion disorders and the neuroprotective properties by pharmacological inhibition or genetic deletion of TXNIP following cerebrovascular and neurodegenerative diseases (Nasoohi et al., 2018; Pan et al., 2018). Taken together, the remarkable overlapped DEGs might be the most potential candidates for the researches on pain-, anxiety-, or depression-disorders.

Despite that we reported the unique transcriptome profiles and conducted a series of functional analyses, the present study still has some limitations. Firstly, it should be noted that the SC, ACC, and AMY contain a variety of cell populations including different types of neurons, astrocytes, and microglia cells. However, all bioinformatics analyses presented in this work were obtained from all cell populations. Thus, future studies on the cell-type-specific changes in gene expression following peripheral nerve injury should be performed using single-cell sequencing analysis. Secondly, we used the database of anxiety- and depression-related genes to analyze the DEGs in the ACC and AMY after SNL. However, six days post-SNL, anxiety- and depression-like behaviors were not completely developed even if SNL-induced aversion was detected (Suzuki et al., 2007; Wu et al., 2019). However, the evidence demonstrated that the analyzed anxiety- and depression-related DEGs such as Gria1 (Rivera et al., 2020), Mapk1 (Sierra-Fonseca et al., 2019), Mapk9 (Thomson et al., 2020), and Fkbp5 (Zannas et al., 2019) contributed to anxiety or depression symptoms in rodents. The further study on the gene expression profiles and emotion-related behaviors including anxiety- and depression-like behaviors at the later stage of neuropathic pain should be carried out. Thirdly, investigations on the gender-specific and age-specific pain mechanisms should also be included due to the more frequent prevalence of pain in women and aged patients (Bouhassira et al., 2008). Fourthly, other brain regions such as the medial prefrontal cortex, nucleus accumbens, and periaqueductal gray were reported to participate in pain- and emotion-related behaviors under the conditions of chronic stress and/or chronic pain as well (Bouhassira et al., 2008; Descalzi et al., 2017; Smith et al., 2021). To obtain more valuable information, RNA sequencing analysis at these brain regions needs to be considered in the future. Finally, although the present study demonstrated gene transcript alternations and their functional analyses in the SC, ACC, and AMY, whether these changes contribute to the induction and maintenance of neuropathic pain and whether they can serve as new targets remain to be further determined.

## Conclusion

In summary, we for the first time provided the unique gene expression profiles of lncRNAs and mRNAs in three pain-related regions and revealed the implication of neuroinflammation and apoptosis in the pathogenesis of neuropathic pain using different bioinformatics analyses. The comparisons of RNA sequencing results provide a more thorough analysis of gene expression alterations in three distinct pain-related regions. Overall, our findings present comprehensive information that may facilitate the discovery of novel analgesic strategies.

## Data Availability Statement

The datasets presented in this study can be found in online repositories. The name of the repository and accession number can be found below: National Center for Biotechnology Information (NCBI) BioProject, https://www.ncbi.nlm.nih.gov/bioproject/, PRJNA705299.

## Ethics Statement

The animal study was reviewed and approved by the Animal Care and Use Committee of Zhengzhou University.

## Author Contributions

WZ conceived the project, supervised all the experiments, and edited the manuscript. SS, ML, DW, JC, XR, Y-XT, and WZ assisted with experimental design. SS and ML carried out behavioral tests, surgery, and tissue collection. SS performed the RT-PCR assay and wrote the draft of the manuscript. SS, ML, and DW analyzed the data. Y-XT and WZ edited the manuscript. All authors read and discussed the manuscript.

## Conflict of Interest

The authors declare that the research was conducted in the absence of any commercial or financial relationships that could be construed as a potential conflict of interest.

## Footnotes

1 http://www.omicshare.com/tools

2 https://www.genecards.org/

3 http://ctdbase.org/

4 https://bioinfogp.cnb.csic.es/tools/venny/index.html

5 http://david.abcc.ncifcrf.gov/

6 http://www.geneontology.org

7 http://www.genome.jp/kegg/

8 https://string-db.org/cgi/

9 www.cytoscape.org/

## Supplementary Material

Supplementary Figure 1 Transcriptome profiling in the SC, ACC and AMY in mice after nerve injury. **(A)** The mapped proportions of exonic, intronic, and intergenic reads in the SC, ACC and AMY on day 7 post-SNL or sham surgery. **(B-D)** The proportions of reads that align to exonic, intronic and intergenic regions from the SC **(B)**, ACC **(C)**, and AMY **(D)** on day 7 post-surgery. *n* = 3 biological repeats. ^∗^*P* < 0.05; ^∗∗^*P* < 0.01 versus the corresponding sham group by two-tailed unpaired Student’s *t*-test. **(E-G)** Distribution of differentially expressed RNAs in SC **(E)**, ACC **(F)**, and AMY **(G)** on day 6 after SNL.

Supplementary Figure 2 Heatmaps of the representative differentially expressed genes (DEGs) in the ACC after nerve injury. Top 15 up-and down-regulated G protein-coupled receptor mRNAs **(A)**, ion channel mRNAs **(B)**, and lncRNAs **(C)** in the ACC on day 7 after SNL. Colors in the heatmaps indicate the Row Z-score among the different datasets. The up- and down-regulated genes are colored in red and green, respectively.

Supplementary Figure 3 Heatmaps of the representative differentially expressed genes (DEGs) in the AMY after nerve injury. Top 15 up- and down-regulated G protein-coupled receptor mRNAs **(A)**, ion channel mRNAs **(B)**, and lncRNAs **(C)** in the AMY on day 7 after SNL. Colors in the heatmaps indicate the Row Z-score among the different datasets. The up- and down-regulated genes are colored in red and green, respectively.

Supplementary Figure 4 The common biological processes and KEGG pathways in the SC, ACC and AMY after nerve injury. **(A-D)** The top 10 common enrichments in biological processes (blue panels on the left) and KEGG pathways (red panels on the right) between SC and ACC **(A)**, SC and AMY **(B)**, or ACC and AMY **(C)**. The top 10 common function enrichments in biological processes (blue panels on the left) and KEGG pathways (red panels on the right) among SC, ACC and AMY **(D)** according to the *P*-value. Blue and red bars represent the biological process and pathway enrichments, respectively.

Supplementary Figure 5 Heatmaps of the identified pain-, anxiety- and depression-related genes in the SC, ACC and AMY after nerve injury. **(A)** Heatmaps of 157 (100 upregulated and 57 downregulated) pain-, 220 (140 upregulated and 80 downregulated) anxiety- and 278 (176 upregulated and 102 downregulated) depression-related DEGs in the ACC. **(B)** Heatmaps of 149 (100 upregulated and 49 downregulated) pain-, 201 (146 upregulated and 55 downregulated) anxiety- and 287 (207 upregulated and 80 downregulated) depression-related DEGs in the AMY; ***C*,** Heatmaps of 230 (196 upregulated and 34 downregulated) pain-related DEGs in the spinal cord. Colors in the heatmaps indicate the Row Z-score among the different datasets. The up- and down-regulated genes are colored in red and green, respectively.

Supplementary Figure 6 The functional prediction of DE lncRNAs by GO and KEGG analyses in the ACC from SNL mice. **(A-C)** Directed Acyclic Graphs (DAGs) graphically display the significant GO enrichment results with the candidate targeted genes in biological process **(A)**, molecular function **(B)**, and cellular component **(C)**. **(D)** The significant molecular function, biological process and cellular component enrichment analysis of DE lncRNA-related mRNAs. **(E,F)** The DE lncRNA-related mRNAs enriched KEGG pathways represented by enrichment scores (−log10 (*P*-value)) **(E)** and the scatterplot showing statistics of pathway enrichment **(F)**, respectively. The color of pathway terms is defined by the enrichment *P*-value.

Supplementary Figure 7 The functional prediction of DE lncRNAs by GO and KEGG analyses in the AMY from SNL mice. **(A-C)** Directed Acyclic Graphs (DAGs) graphically display the significant GO enrichment results with the candidate targeted genes in biological process **(A)**, molecular function **(B)**, and cellular component **(C)**. **(D)** The significant molecular function, biological process and cellular component enrichment analysis of DE lncRNA-related mRNAs. **(E,F)** The DE lncRNA-related mRNAs enriched KEGG pathways represented by enrichment scores (−log10 (*P*-value)) **(E)** and the scatterplot showing statistics of pathway enrichment **(F)**, respectively. The color of pathway terms is defined by the enrichment *P*-value.
