# Supplementary material for: Gene Transcript Alterations in the Spinal Cord, Anterior Cingulate Cortex, and Amygdala in Mice Following Peripheral Nerve Injury
Source: Front Cell Dev Biol. 2021 Apr 7;9:634810. doi: 10.3389/fcell.2021.634810 (PMC8059771; doi:10.3389/fcell.2021.634810)
Supplement: Supplementary file 1 [file Table_1.DOCX]

Supplementary Table 1 overlapped genes between pain and neuroinflammation related genes

| Neuroinflammation and apotosis related genes | Pain related genes in different regions | | |
| --- | --- | --- | --- |
|  | SC | ACC | AMY |
| Apoptosis | Atf3, Aifm1, Casp12, Eif4ep1 Max, Slc4a1 | Axl, E2f1, Gli1 | Aifm1, Cdkn1c, Daxx |
| Inflammation | Col7a1, Dcn, Nf2, Hrh1, Ibsp, Pla2g4a | Hrh1, Nf2 | Adcyap1, Trpm8 |
| Immunity | Alad, Cd68, Cdkn2c, Cebpd，Cfl1, Clcn7, Deaf1, Dlg4，Eif4g1, Fhl1, Hexb, Il31ra, Isg15, Lifr, Osmr, Pld1, Pmp22，Pygl, Slc2a2, Stim1, Trps1, Trrobp, Ung | Alad, Cdsn, Chat, Gnb3, Oprk1, Phex, Pygl, Trps1, Usp8 | Aif1, Alad, Deaf1, Dnm2 Eif4g1, Il31ra, Matr3, Oprk1, Orm1, Phex, Stim1, stxbp1, Tcirg1 |
| Apoptosis and Inflammation | Lipe, Fhit, Sparc, | Pcks9, Postn, Sparc | Lipe, Fhit, Sparc, Postin |
| Apoptosis and Immunity | Bak1, Birc2, Casp4, Cdk1, Ccnd3, Limk1, Ifl27, Shc1, Spta1, Trem2, Ube3a | Abl1, Bak1, Ccnd3, Diablo, Dnmt3b, Mpl | Bad, Ccnd3, Cyp17a1, Diablo, Ewsr1, Ube3a |
| Inflammation and Immunity | Acadvl, Acp5, Aprt, C1qa, Cacna1c, Capn3, Ccr1, Cp, Crlf1, Cyba, F13a1, Gal, Gba, Ikzf1, Il12rb1, Lcn2, Matn3, Mb, Mt-nd6, Mvk, Ncf1, Scn9a, Serpinc1, Serping1, Sh2b3, Slc2a4, Tcf4, Tlr7, Trex1, Txnip, Ube2l3, Xdh | Acadvl, Ano5, Apoh, Cacna1c, Camk2d, Ccr2, Dnase1l3, Etv6, F5, Fip1l1, Ikzf1, Il12a, Kl,Ibp, Mpv17, Mpz, Prkar1a, Sh2b3, Slc17a5, Slpi, Tcf4, Txnip | Acadvl, Acp5, Adam17, Ano5, Atxn2, Cacna1c, Camk2d, Col1a2, Crlf1, Cybb, F13a1, Fip1l1, Gad1, Htr3a, Il33, Irak1, Map2k6, Ncstn, Nr1h3, Capn3, Hspg2, Kng1, Ncf1, Nr1i2, Tcf4 |
| Apoptosis, Inflammation and Immunity | Abcc2, Apoe, Areg, Aurka, B2m, Bax, Bcl2l1, Bdnf, C3, Casp3, Casp9, Ccl2, Ccr5, Ccr6, Cd14, Cd274, Cd34, Cd44, Cdk2, Cdkn2a, Cdkn2b, Cdkn3, Cfh, Col1a1, Comp, Crh, Csf1, Ctsd, Cx3cr1, Cxcl10, Cyp1b1, Des, Elane, Emd, Epo, Fbn1, Fgfr1, Fgfr2, Fgfr3, Flnc, Fn1, Foxp3, Fst, Gapdh, Gfap, Ggt1, Gnas, Grn, Gsk3b, Havcr2, Hgf, Hmgcr, Icam1, Igf1, Igf2, Il15, Il1a, Il6st, Irf1, Itgam, Itgb2, Lgals1, Mapk8, Men1, Mfn2, Mlh1, Mmp3, Mpo, Nfkb1, Ngfr, Npy, Nras, Ntrk2, Pecam1, Plau, Plaur, Pml, Polg, Psmb8, Ptgs2, Runx2, S100a8, Scn1a, Sirt1, Socs3, Stat1, Stat3, Tardbp, Tgfbr2, Tlr2, Tlr3, Tlr9, Tnfrsf1b, Tnfsf11, Tsc1, Tspo, Vim, Vip | Abcc1, Abcg2, Aurka, C3,  Casp3, Ccr6, Cd4, Cfh, Chuk, Cryab, Ddit3, Dnm1l, Drd1, Elane, Epcam, Fgfr3, Flna, Fmr1, Fn1, Gdnf, Ggt1, Gnas, Hfe, Igf1, Il1r1, Il1rn, Il2rb, Kmt2a, Ipl, Mapk1, Mapk3, Mapk8, Mapk9, Mdm2, Men1, Mme, Myc, Myh7, Nfkb1, Nos1, Nr3c1, Plaur, Pth1r, Rac1, Runx1, Runx2, Sell, Serpine1, Spp1, Tac1, Tardbp, Tcf7l2, Ttr, Xiap | Abcg2, Apob, Aurka, Bscl2, C3, Casp3, Casp8, Cck, Cd44, Cd8a, Cdh1, Cdkn1a, Cfh, Cflar, Cr2, Crh, Cryab, Cxcl10, Cxcl12, F2, Fgfr1, Fgfr3, Frzb, Gast, Ggt1, Gnas, Hgf, Htr2a, Icam1, Igf2, Il15, Il5, Itgam, Kcnh2, Lgals1, Mapk1, Mapk14, Men1, Myc, Nog, Nos1, Nr3c1, Nts, Prkcd, Psen1, Scn1a, Sell, Socs3, Spp1, Tardbp, Tcf7l2, Tek, Tnf, Vim, Vip, Vldlr, Xiap |

,
