# Supplementary material for: Gene Transcript Alterations in the Spinal Cord, Anterior Cingulate Cortex, and Amygdala in Mice Following Peripheral Nerve Injury
Source: Front Cell Dev Biol. 2021 Apr 7;9:634810. doi: 10.3389/fcell.2021.634810 (PMC8059771; doi:10.3389/fcell.2021.634810)
Supplement: Supplementary file 2 [file Table_2.DOCX]

Supplementary Table 2: overlapped genes between emotion disorders related genes and neuroinflammation related genes

| Neuroinflammation related genes | Emotion disorders related genes in different regions | | | |
| --- | --- | --- | --- | --- |
|  | ACC | | AMY | |
|  | Anxiety | Depression | Anxiety | Depression |
| Apoptosis | Htra2 | E2f1, Gli1, Satb1,  Tfdp2 | Aifm1, Aurkb，Cdkn1c, Tubab | Aifm1, Aurkb，Cdkn1c, Rassf1, Satb1,Tfdp2, Tubab, Unc5b |
| Inflammation | A2m, Arg2, Lpin1,  Hrh1, Kcnma1 | A2m, Arg2, Lpin1,  Hrh1 | Adcyap1, Kcnma1 | Adcyap1 |
| Immunity | Acly, Actn2, Adnp, Alad, Chat, Cpne1, Foxp1, Iqsec2, Mef2c, Nedd4l, Nsd2, Oprk1, Pcdh19, Racgap1, Socs2, Ube2c, Usp8 | Acly, Adnp, Alad, Asb7, Atp6v0c Cd83, Cdc34, Chat, Crk, Foxp1, Foxp2, Gnb3, Grin2c, Inppl1, Iqsec2, Kdm6a, Lmo7, Mef2c, Msr1, Nedd4l, Nrxn1, Nsd2, Pld3, Pygl, Racgap1, Sec31a, Socs2, Tgif1, Ube2c, Ube2d3 | Aif1, Alad, Cd74，Col4a1,Cpne1, Deaf1, Ehmt1, Eif4g1, Gtf2i, Hspa1b, Mef2c, Nedd4l, Nsd2, Oprk1, Orm1, Pcdh19, Prkar2b, Ptpn1, Ptprj, Ptprn1, Rrs6ka3, Uba1, Ube2c | Aif1, Alad, Asb7, Atp6v1a, Blnk, C1qc, Cd74, Cd83, Cdc34, Col4a1, Creg1, Crk, Deaf1, Disc1, Ehmt1, Eif4g1, Fgl2, Foxp2, Hspa1b, Mef2c, Mrc1, Msr1, Nedd4l, Nrxn1, Nsd2, Orm1, Psap, Rnf4, Scamp1, Sec31a, Sin3a, Snap25, Stbd1, Stxbp1, Tgif1, Ube2c |
| Apoptosis and Inflammation | Pcks9, Sparc | Pcks9, Postn, Sparc | Lipe, Sparc | Lipe, Postn , Sparc |
| Apoptosis and Immunity | Bak1, Ccnd3, Dnmt3b | Bak1, Ccnd3, Diablo, Dnmt3b, Myb, Ntrk3，Tnfrsf25 | Bad, Ccnd3, Cyp17a1, Hnf1a, Psmd2, Ube3a | Actg1, Bad, Bcl3, Ccnd3, Cyp17a1, Diablo, Pak1, Ube3a |
| Inflammation and Immunity | Cacna1c, Camk2b, Camk2d, Ccr2, cdh23, Il12a, Lbp, Mbp, Mlxipl, Nr4a2, Prkar1a, Txnip | Cacna1c, Camk2b Camk2d, Ccr2, Ido1, Il12a, Ibp, Mbp, Nr4a2, Prkar1a, Slc17a5, Tcf4, Tmem216, Txnip | Acp5, Adam17, Arg1, Atxn2, Cacna1c, Camk2a, Camk2b, Col1a2, Crlf1, Cybb, Gad1, Htr3a, Il33, Map2k6, Kng1, Ncf1, Nr1i2, Nr4a2 | Acp5, Adam17, Arg1, Atxn2, Cacna1c, Camk2a, Camk2b, Col1a2, Crlf1, Cybb, F13a1, Fcgr2b, Gad1, Hspg2, Htr3a, Il33, Irak1, Map2k6, Kng1, Ncf1, Nr1h3, Nr1i2, Nr4a2, Rtn4，Tcf4, |
| Apoptosis, Inflammation and Immunity | Abcc1, Abcg2, Aurka,  C3, Casp3, Cfh, Cryab, Ddit3, Dnm1l, Drd1, Epcam, Flna, Fmr1, Fn1, Fyn, Gdnf, Ghr, Gnas, Hfe, Igf1, Il1rn, Keap1, Krt18, Ipl, Mapk1, Mapk10, Mapk3, Mapk8, Mapk9, Mdm2, Men1, Mme, Myc, Myh7, Nfkb1, Nos1, Nr3c1, Nrg1, Plec, Rac1, Retn, Runx2, Serpine1, Spp1, Tac1, Tardbp, Ttr, Xiap | Abcc1, Abcg2, Atg7, Aurka, C3, Casp3, Cd4, Cfh, Chuk, Cryab, Ddit3, Dnm1l, Drd1, Dst, Epcam, Fgfr3, Flna, Fmr1, Fn1, Fyn，Gdnf, Ggt1, Gnas, Ghr，Hfe，Igf1, Il1r1, Il1rn, Keap1, Kmt2a, Krt18, Ipl, Mapk1, Mapk3,Mapk8，Mapk9, Mapk10,Mdm2, Mme, Myc, Myh7, Nfkb1, Nos1, Nr3c1, Nrg1, Otx2, Pax6, Plaur, Plec，Pth1r, Rac1, Rarb, Rten, Runx1, Runx2, Serpine1, Socs1, Spp1, Tac1, Tcf7l2, Tardbp, Ttr, Xiap | Abcg2, Apob, Aurka, C3, Cartpt, Casp3, Casp8, Cck, Cd44, Cdh1, Cdkn1a, Cfh, Col6a3, Crh, Cryab, Csf1r, Cxcl10,Cxcl12, F2, Fgfr1, Gnas, Grb2, Htr2a, Icam1, Igf2, Il5, Itgam, Kcnh2, Keap1，Lgals1, Mapk1, Mapk14, Men1, Myc, Nos1, Nr3c1, Nts, Plec, Prkcd, Prom1，Psen1, Rbp4, Scn1a, Socs3, Spp1, Sptan1, Tardbp, Tnf, Vldlr, Xiap | Abcg2, Apob, Aurka  C3, Cartpt, Casp3, Casp8, Cck, Cd44, Cd8a, Cdh1, Cdkn1a, Cfh, Cflar, Col6a3, Crh, Cryab, Csf1r, Cxcl10, Cxcl12, F2, Fgfr1, Fgfr3, Ggt1, Gnas, Hgf, Htr2a, Icam1, Igf2, Il5, Itgam, Igtb4, Kcnh2, Keap1, Lgals1, Mapk1, Mapk14, Myc, Nos1, Nr3c1, Nts, Pax6，Pgf，Plec, Prkcd, Prom1, Psen1, Rbp4, Scn1a, Socs3, Spp1, Tardbp, Tcf7l2, Tek, Tnf, Vim, Vldlr, Xiap |
