# Supplementary material for: Gene Transcript Alterations in the Spinal Cord, Anterior Cingulate Cortex, and Amygdala in Mice Following Peripheral Nerve Injury
Source: Front Cell Dev Biol. 2021 Apr 7;9:634810. doi: 10.3389/fcell.2021.634810 (PMC8059771; doi:10.3389/fcell.2021.634810)
Supplement: Supplementary file 3 [file Table_3.DOCX]

Supplementary Table 3: Overlapped pain related genes across three regions in present study.

| Index | Gene symbol | Description | Regions (Fold change) SNL versus sham | | |
| --- | --- | --- | --- | --- | --- |
|  |  |  | SC | ACC | AMY |
| 1 | Abcg2 | ATP binding cassette subfamily G member 2 (Junior blood group) |  | 0.57 | 2.59 |
| 2 | Acadvl | acyl-CoA dehydrogenase very long chain | 1.80 |  | 3.16 |
| 3 | Acp5 | acid phosphatase 5, tartrate resistant | 21.36 |  | 7.32 |
| 4 | Aifm1 | apoptosis inducing factor mitochondria associated 1 | 1.85 |  | 3.89 |
| 5 | Alad | aminolevulinate dehydratase | 12.40 | 0.063 | 1.79 |
| 6 | Ank1 | ankyrin 1 | 71.30 | 0.14 | 4.65 |
| 7 | Ano5 | anoctamin 5 |  | 0.027 | 2.25 |
| 8 | Antxr2 | ANTXR cell adhesion molecule 2 | 1.77 |  | 0.15 |
| 9 | Anxa4 | annexin A4 | 0.20 | 0.36 |  |
| 10 | Arnt | aryl hydrocarbon receptor nuclear translocator | 5.57 | 0.31 |  |
| 11 | Aurka | aurora kinase A | 10.61 | 4.18 | 0.18 |
| 12 | Bak1 | BCL2 antagonist/killer 1 | 2.27 | 0.52 |  |
| 13 | Bcor | BCL6 corepressor |  | 2.54 | 3.09 |
| 14 | C3 | complement C3 | 1.82 | 3.50 | 2.81 |
| 15 | Cacna1b | calcium voltage-gated channel subunit alpha1 B | 0.34 | 0.50 |  |
| 16 | Cacna1c | calcium voltage-gated channel subunit alpha1 B | 2.05 | 6.46 | 380.17 |
| 17 | Cacna2d1 | calcium voltage-gated channel auxiliary subunit alpha2delta 1 |  | 1.94 | 2.99 |
| 18 | Capn3 | calpain 3 | 2.03 | 0.18 |  |
| 19 | Casp3 | caspase 3 | 3.98 | 5.49 | 3.16 |
| 20 | Ccnd3 | cyclin D3 | 0.02 | 13.33 | 100.03 |
| 21 | Ccr6 | C-C motif chemokine receptor 6 | 7.53 | 0.38 |  |
| 22 | Cd44 | CD44 molecule (Indian blood group) | 2.15 |  | 0.38 |
| 23 | Cfh | complement factor H | 4.18 | 4.96 | 13.68 |
| 24 | Cnbp | CCHC-type zinc finger nucleic acid binding protein | 2.50 | 0.35 |  |
| 25 | Crem | cAMP responsive element modulator | 6.86 | 4.37 | 1.99 |
| 26 | Crh | corticotropin releasing hormone | 2.59 |  | 2.38 |
| 27 | Crlf1 | cytokine receptor like factor 1 | 2.03 |  | 2.67 |
| 28 | Cryab | crystallin alpha B |  | 0.03 | 8.73 |
| 29 | Cxcl10 | C-X-C motif chemokine ligand 10 | 3.72 |  | 3.34 |
| 30 | Deaf1 | DEAF1 transcription factor | 1.75 |  | 2.22 |
| 31 | Diablo | diablo IAP-binding mitochondrial protein |  | 4.11 | 0.44 |
| 32 | Eif4g1 | eukaryotic translation initiation factor 4 gamma 1 | 1.89 |  | 2.27 |
| 33 | Elane | elastase, neutrophil expressed | 4.62 |  | 6.24 |
| 34 | F13a1 | coagulation factor XIII A chain | 2.01 |  | 1.77 |
| 35 | Fgfr1 | fibroblast growth factor receptor 1 | 1.87 |  | 3.57 |
| 36 | Fgfr3 | fibroblast growth factor receptor 3 | 2.17 |  | 2.62 |
| 37 | Fhit | fragile histidine triad diadenosine triphosphatase | 2.42 |  | 11.57 |
| 38 | Fip1l1 | factor interacting with PAPOLA and CPSF1 |  | 0.05 | 6.69 |
| 39 | Flcn | Folliculin |  | 7.00 | 0.33 |
| 40 | Fn1 | fibronectin 1 | 1.85 | 37.86 | 1.63 |
| 41 | Gabra1 | gamma-aminobutyric acid type A receptor subunit alpha1 |  | 0.52 | 0.19 |
| 42 | Ggt1 | gamma-glutamyltransferase 1 |  | 5.18 | 0.058 |
| 43 | Gnas | GNAS complex locus | 8.58 |  | 3.31 |
| 44 | Hgf | hepatocyte growth factor | 2.00 | 0.61 | 2.31 |
| 45 | Homer1 | homer scaffold protein 1 |  | 1.82 | 1.94 |
| 46 | Hrh1 | histamine receptor H1 | 0.19 | 0.50 |  |
| 47 | Icam1 | intercellular adhesion molecule 1 | 2.22 |  | 1.80 |
| 48 | Id2 | inhibitor of DNA binding 2 | 0.07 | 45.86 |  |
| 49 | Igf1 | insulin like growth factor 1 | 7.36 | 0.16 |  |
| 50 | Igf2 | insulin like growth factor 2 | 3.86 |  | 2.13 |
| 51 | Ikzf1 | IKAROS family zinc finger 1 | 11.24 | 8.18 |  |
| 52 | Il15 | interleukin 15 | 4.23 |  | 10.64 |
| 53 | Il31ra | interleukin 31 receptor A | 2.21 |  | 3.43 |
| 54 | Itgam | integrin subunit alpha M | 4833 |  | 2.21 |
| 55 | Kif1a | kinesin family member 1A | 0.16 |  | 0.28 |
| 56 | Lgals1 | galectin 1 | 1.81 |  | 1.78 |
| 57 | Lipe | lipase E, hormone sensitive type | 0.44 |  | 0.03 |
| 58 | Litaf | lipopolysaccharide induced TNF factor | 0.18 | 5.02 |  |
| 59 | Mapk1 | mitogen-activated protein kinase 1 |  | 1.94 | 1.77 |
| 60 | Mapk8 | mitogen-activated protein kinase 8 | 1.80 | 0.10 |  |
| 61 | Men1 | menin 1 | 9.25 | 18.17 | 4.30 |
| 62 | Mtm1 | myotubularin 1 | 5.32 | 0.24 | 0.43 |
| 63 | Myc | MYC proto-oncogene, bHLH transcription factor |  | 0.24 | 2.47 |
| 64 | Ncf1 | neutrophil cytosolic factor 1 | 114.45 |  | 0.11 |
| 65 | Nf2 | neurofibromin 2 | 0.38 | 0.20 |  |
| 66 | Nfkb1 | nuclear factor kappa B subunit 1 | 28.46 | 0.27 |  |
| 67 | Nos1 | nitric oxide synthase 1 |  | 9.87 | 0.53 |
| 68 | Nr1i3 | nuclear receptor subfamily 1 group I member 3 | 6.88 |  | 0.09 |
| 69 | Nr3c1 | nuclear receptor subfamily 3 group C member 1 |  | 0.38 | 3.96 |
| 70 | Nrf1 | nuclear respiratory factor 1 | 3.80 | 0.15 | 0.53 |
| 71 | Oprk1 | opioid receptor kappa 1 |  | 7.00 | 2.83 |
| 72 | Oprl1 | opioid related nociceptin receptor 1 | 1.94 | 11.31 | 3.45 |
| 73 | Pdyn | Prodynorphin | 2.01 | 1.97 |  |
| 74 | Phex | phosphate regulating endopeptidase homolog X-linked |  | 2.60 | 3.21 |
| 75 | Plaur | plasminogen activator, urokinase receptor | 8.56 | 3.31 |  |
| 76 | Pln | Phospholamban |  | 0.37 | 0.11 |
| 77 | Postn | Periostin |  | 5.25 | 0.39 |
| 78 | Ppp1r1b | protein phosphatase 1 regulatory inhibitor subunit 1B | 2.23 | 3.54 |  |
| 79 | Pygl | glycogen phosphorylase L | 1.88 | 2.30 |  |
| 80 | Runx2 | RUNX family transcription factor 2 | 0.18 | 4.29 |  |
| 81 | Scn1a | sodium voltage-gated channel alpha subunit 1 | 0.39 | 0.71 | 0.22 |
| 82 | Sell | selectin L |  | 7.23 | 6.39 |
| 83 | Sgk1 | serum/glucocorticoid regulated kinase 1 | 0.27 | 0.29 |  |
| 84 | Sh2b3 | SH2B adaptor protein 3 | 14.11 | 2.07 |  |
| 85 | Socs3 | suppressor of cytokine signaling 3 | 2.70 |  | 1.91 |
| 86 | Sparc | secreted protein acidic and cysteine rich | 3.39 | 3.05 | 3.44 |
| 87 | Spp1 | secreted phosphoprotein 1 |  | 6.13 | 0.33 |
| 88 | Stim1 | stromal interaction molecule 1 | 31.55 |  | 2.21 |
| 89 | Tardbp | TAR DNA binding protein | 2.88 | 0.42 | 19.99 |
| 90 | Tcf4 | transcription factor 4 | 11.53 | 0.35 | 0.14 |
| 91 | Tcf7l2 | transcription factor 7 like 2 |  | 66.54 | 0.51 |
| 92 | Tpm1 | tropomyosin 1 | 0.45 | 4.89 |  |
| 93 | Trappc2 | trafficking protein particle complex 2 | 4.38 |  | 2.70 |
| 94 | Trp53 | transformation related protein 53 | 3.25 | 4.05 | 2.69 |
| 95 | Trps1 | transcriptional repressor GATA binding 1 | 0.30 | 2.15 |  |
| 96 | Tsc22d3 | TSC22 domain family member 3 | 2.15 |  | 2.10 |
| 97 | Txnip | thioredoxin interacting protein | 2.76 | 1.86 | 1.72 |
| 98 | Ube3a | ubiquitin protein ligase E3A | 33.69 |  | 3.77 |
| 99 | Vgf | VGF nerve growth factor inducible | 1.75 |  | 2.17 |
| 100 | Vim | Vimentin | 2.24 |  | 1.91 |
| 101 | Vip | vasoactive intestinal peptide | 2.23 |  | 2.80 |
| 102 | Wnk1 | WNK lysine deficient protein kinase 1 | 0.65 | 2.22 | 2.11 |
| 103 | Xiap | X-linked inhibitor of apoptosis |  | 3.56 | 0.24 |
