# Supplementary material for: Gene Transcript Alterations in the Spinal Cord, Anterior Cingulate Cortex, and Amygdala in Mice Following Peripheral Nerve Injury
Source: Front Cell Dev Biol. 2021 Apr 7;9:634810. doi: 10.3389/fcell.2021.634810 (PMC8059771; doi:10.3389/fcell.2021.634810)
Supplement: Supplementary file 4 [file Table_4.DOCX]

Supplementary Table 4: Overlapped genes related to pain, anxiety and depression in ACC and AMY following peripheral nerve injury

| Index | Gene name | Regions SNL versus sham (Fold change) | | Associated disorders |
| --- | --- | --- | --- | --- |
|  |  | ACC | AMY |  |
| 1 | A2m | 2.05 |  | anxiety, depression |
| 2 | Abcc1 | 10.05 |  | pain, anxiety, depression |
| 3 | Abcc5 | 2.86 |  | pain, depression |
| 4 | Abcg2 | 0.57 | 2.59 | pain, anxiety, depression |
| 5 | Abhd4 |  | 0.45 | anxiety, depression |
| 6 | Ablim1 | 1.90 | 2.20 | anxiety, depression |
| 7 | Acacb | 4.20 |  | anxiety, depression |
| 8 | Acly | 0.02 |  | anxiety, depression |
| 9 | Acp5 |  | 7.32 | pain, anxiety, depression |
| 10 | Acss2 |  | 0.56 | anxiety, depression |
| 11 | Adam17 |  | 3.34 | pain, anxiety, depression |
| 12 | Adcyap1 |  | 2.86 | pain, anxiety, depression |
| 13 | Adnp | 0.38 |  | anxiety, depression |
| 14 | Adra2a |  | 1.87 | pain, anxiety, depression |
| 15 | Aif1 |  | 0.32 | pain, anxiety, depression |
| 16 | Aifm1 |  | 3.89 | pain, anxiety, depression |
| 17 | Alad | 0.06 | 1.79 | pain, anxiety, depression |
| 18 | Alas2 |  | 2.35 | anxiety, depression |
| 19 | Ank1 | 0.14 | 4.65 | pain, anxiety, depression |
| 20 | Ano3 |  | 0.48 | pain, anxiety |
| 21 | Anxa3 | 2.09 |  | pain, anxiety, depression |
| 22 | Anxa4 |  | 0.36 | pain, depression |
| 23 | Apob |  | 0.26 | pain, anxiety, depression |
| 24 | Aqp1 | 5.05 |  | anxiety, depression |
| 25 | Arc | 0.64 |  | pain, anxiety, depression |
| 26 | Arg1 |  | 0.48 | anxiety, depression |
| 27 | Arg2 | 1.74 |  | anxiety, depression |
| 28 | Arhgap26 |  | 2.55 | anxiety, depression |
| 29 | Arnt | 0.31 |  | pain, anxiety, depression |
| 30 | Arrdc3 | 4.98 | 0.57 | anxiety, depression |
| 31 | Asph |  | 0.45 | anxiety, depression |
| 32 | Atp2b4 |  | 1.97 | anxiety, depression |
| 33 | Atxn2 | 1.68 | 1.88 | pain, anxiety, depression |
| 34 | Aurka | 4.18 | 0.18 | pain, anxiety, depression |
| 35 | Aurkb |  | 6.17 | anxiety, depression |
| 36 | Avp | 14.72 |  | pain, anxiety, depression |
| 37 | Avpr1b | 11.02 |  | anxiety, depression |
| 38 | Bad |  | 1.83 | pain, anxiety, depression |
| 39 | Bak1 | 0.51 |  | pain, anxiety, depression |
| 40 | Bdh1 | 0.02 |  | pain, depression |
| 41 | Blvrb | 3.05 |  | anxiety, depression |
| 42 | Btg2 | 0.57 |  | pain, anxiety, depression |
| 44 | C3 | 3.50 | 2.81 | pain, anxiety, depression |
| 45 | Cacna1c | 6.46 | 380.17 | pain, anxiety, depression |
| 46 | Cacna1h |  | 0.66 | pain, anxiety, depression |
| 47 | Cacna2d1 | 1.94 | 2.99 | pain, depression |
| 48 | Camk2a |  | 2.77 | pain, anxiety, depression |
| 49 | Camk2b | 2.66 | 4.22 | anxiety, depression |
| 50 | Camk2d | 0.11 |  | pain, anxiety, depression |
| 51 | Cap2 | 1.98 |  | anxiety, depression |
| 52 | Cartpt |  | 1.99 | anxiety, depression |
| 53 | Casp3 | 5.49 | 3.16 | pain, anxiety, depression |
| 54 | Casp8 |  | 1.79 | pain, anxiety, depression |
| 55 | Cav2 | 0.43 |  | anxiety, depression |
| 56 | Cck |  | 2.01 | pain, anxiety, depression |
| 57 | Ccnd3 | 13.33 | 100.03 | pain, anxiety, depression |
| 58 | Ccng2 | 4.48 | 4.27 | anxiety, depression |
| 59 | Ccr2 | 3.52 |  | pain, anxiety, depression |
| 60 | Cd4 | 6.89 |  | pain, depression |
| 61 | Cd44 |  | 0.38 | pain, anxiety, depression |
| 62 | Cd52 |  | 1.80 | anxiety, depression |
| 63 | Cd74 |  | 3.53 | anxiety, depression |
| 64 | Cd8a |  | 7.37 | pain, depression |
| 65 | Cdc25b | 0.60 |  | anxiety, depression |
| 66 | Cdh1 |  | 10.17 | pain, anxiety, depression |
| 67 | Cdkn1c |  | 16.1 | pain, anxiety, depression |
| 68 | Cfh | 4.96 | 13.68 | pain, anxiety, depression |
| 69 | Cflar |  | 0.42 | pain, depression |
| 70 | Chat | 3.97 |  | pain, anxiety, depression |
| 71 | Chuk | 2.40 |  | pain, depression |
| 72 | Ckm | 0.49 |  | anxiety, depression |
| 73 | Ckmt1 |  | 2.43 | anxiety, depression |
| 74 | Cldn1 | 2.48 |  | anxiety, depression |
| 75 | Clock | 2.23 |  | anxiety, depression |
| 76 | Col11a2 |  | 0.64 | pain, depression |
| 77 | Col1a2 |  | 1.79 | pain, anxiety, depression |
| 78 | Col4a1 |  | 0.50 | anxiety, depression |
| 79 | Col6a3 |  | 1.90 | anxiety, depression |
| 80 | Crem | 4.37 | 1.99 | pain, anxiety, depression |
| 81 | Crh |  | 2.38 | pain, anxiety, depression |
| 82 | Crhr2 | 2.94 |  | anxiety, depression |
| 83 | Crlf1 |  | 2.67 | pain, anxiety, depression |
| 84 | Cryab | 0.03 | 8.73 | pain, anxiety, depression |
| 85 | Csf1r |  | 3.18 | anxiety, depression |
| 86 | Cxcl10 |  | 3.34 | anxiety, depression |
| 87 | Cxcl12 |  | 0.22 | anxiety, depression |
| 88 | Cybb |  | 2.23 | pain, anxiety, depression |
| 89 | Cyp17a1 |  | 0.21 | pain, anxiety, depression |
| 90 | Cyp26b1 | 1.74 | 1.96 | anxiety, depression |
| 91 | Dbn1 | 2.41 |  | anxiety, depression |
| 92 | Dclk1 | 0.56 | 2.33 | anxiety, depression |
| 93 | Dcxr |  | 2.01 | anxiety, depression |
| 94 | Ddit3 | 4.05 |  | pain, anxiety, depression |
| 95 | Deaf1 |  | 2.24 | pain, anxiety, depression |
| 96 | Diablo | 4.11 | 0.44 | pain, depression |
| 97 | Dnaja4 | 2.11 |  | anxiety, depression |
| 98 | Dnm1l | 2.11 |  | pain, anxiety, depression |
| 99 | Dnmt3b | 12.70 |  | pain, anxiety, depression |
| 100 | Drd1 | 9.82 |  | pain, anxiety, depression |
| 101 | Drd5 | 1.84 |  | pain, anxiety, depression |
| 102 | Dusp1 | 0.66 |  | pain, anxiety, depression |
| 103 | Dyrk2 |  | 1.76 | anxiety, depression |
| 104 | E2f1 | 0.44 |  | pain, depression |
| 105 | Ehmt1 |  | 3.02 | anxiety, depression |
| 106 | Eif4g1 | 0.63 | 2.27 | pain, anxiety, depression |
| 107 | Eif5a | 0.20 | 2.56 | anxiety, depression |
| 108 | Emp1 | 2.26 | 0.26 | anxiety, depression |
| 109 | Eno2 | 0.65 |  | pain, anxiety, depression |
| 110 | Enpp2 | 2.34 |  | pain, anxiety, depression |
| 111 | Epcam | 0.11 |  | pain, anxiety, depression |
| 112 | Erbb4 | 0.59 |  | pain, depression |
| 113 | F13a1 |  | 1.77 | pain, depression |
| 114 | F2 |  | 0.38 | pain, anxiety, depression |
| 115 | Fam107a | 0.37 |  | anxiety, depression |
| 116 | Fat1 |  | 0.23 | anxiety, depression |
| 117 | Fbln1 |  | 2.13 | anxiety, depression |
| 118 | Fgd4 |  | 3.73 | pain, anxiety, depression |
| 119 | Fgfr1 |  | 3.57 | pain, anxiety, depression |
| 120 | Fgfr3 | 103.92 | 2.62 | pain, depression |
| 121 | Fhl2 |  | 1.99 | pain, anxiety, depression |
| 122 | Fkbp4 | 0.36 |  | pain, anxiety, depression |
| 123 | Fkbp5 | 11.78 | 3.12 | anxiety, depression |
| 124 | Flna | 2.72 |  | anxiety, depression |
| 125 | Fmo1 | 0.45 |  | pain, anxiety, depression |
| 126 | Fmo5 | 2.61 | 0.33 | anxiety, depression |
| 127 | Fnbp1 | 19.77 | 0.43 | anxiety, depression |
| 128 | Fos | 0.58 |  | pain, anxiety, depression |
| 129 | Foxp1 | 2.41 |  | anxiety, depression |
| 130 | Fyn | 0.46 |  | anxiety, depression |
| 131 | Gabra1 | 0.52 | 0.19 | pain, anxiety, depression |
| 132 | Gad1 |  | 7.94 | pain, anxiety, depression |
| 133 | Gcnt2 | 2.31 | 1.62 | pain, depression |
| 134 | Gdnf | 3.58 |  | pain, anxiety, depression |
| 135 | Ggt1 | 5.18 | 0.05 | pain, depression |
| 136 | Ghr | 0.27 |  | anxiety, depression |
| 137 | Gli1 | 0.53 |  | pain, depression |
| 138 | Gnai2 |  | 0.56 | pain, anxiety, depression |
| 139 | Gnao1 | 2.08 | 1.53 | anxiety, depression |
| 140 | Gnas | 3.09 | 3.31 | pain, anxiety, depression |
| 141 | Gnb3 | 6.44 |  | pain, depression |
| 142 | Gne |  | 0.63 | pain, depression |
| 143 | Gpat3 |  | 3.03 | pain, depression |
| 144 | Gpx4 | 8.40 |  | anxiety, depression |
| 145 | Gria1 | 59.61 | 1.61 | pain, anxiety, depression |
| 146 | Grm1 | 1.98 |  | pain, anxiety, depression |
| 147 | Grm5 | 0.30 |  | anxiety, depression |
| 148 | Gsta3 |  | 0.10 | pain, anxiety, depression |
| 149 | Gstm5 |  | 0.66 | anxiety, depression |
| 150 | Gstt2 |  | 1.82 | anxiety, depression |
| 151 | Hfe | 0.14 |  | pain, anxiety, depression |
| 152 | Hgf | 0.61 | 2.31 | pain, depression |
| 153 | Higd1a | 0.10 |  | anxiety, depression |
| 154 | Hmga1 |  | 2.21 | anxiety, depression |
| 155 | Hnrnpdl | 0.40 |  | anxiety, depression |
| 156 | Hnrnph1 | 1.87 |  | anxiety, depression |
| 157 | Homer1 | 1.82 | 1.94 | pain, anxiety, depression |
| 158 | Hrh1 | 0.50 |  | pain, anxiety, depression |
| 159 | Hs3st2 |  | 2.12 | anxiety, depression |
| 160 | Hsd11b1 | 0.53 |  | pain, anxiety, depression |
| 161 | Hspa1b |  | 1.79 | anxiety, depression |
| 162 | Hspg2 |  | 0.27 | pain, depression |
| 163 | Htr2a |  | 1.78 | pain, anxiety, depression |
| 164 | Htr3a |  | 1.74 | pain, anxiety, depression |
| 165 | Icam1 |  | 1.80 | pain, anxiety, depression |
| 166 | Id2 | 45.86 |  | pain, anxiety, depression |
| 167 | Igf1 | 0.16 |  | pain, anxiety, depression |
| 168 | Igf2 |  | 2.13 | pain, anxiety, depression |
| 169 | Igfbp4 |  | 1.78 | anxiety, depression |
| 170 | Il12a | 0.09 |  | pain, anxiety, depression |
| 171 | Il1r1 |  | 2.25 | pain, depression |
| 172 | Il1rn | 8.03 |  | pain, anxiety, depression |
| 173 | Il33 |  | 7.26 | pain, anxiety, depression |
| 174 | Il5 |  | 0.09 | pain, anxiety, depression |
| 175 | Iqsec2 | 8.46 |  | anxiety, depression |
| 176 | Irak1 |  | 1.86 | pain, depression |
| 177 | Itga5 | 4.16 |  | anxiety, depression |
| 178 | Itgam |  | 2.21 | pain, anxiety, depression |
| 179 | Kcnh2 |  | 0.28 | pain, anxiety, depression |
| 180 | Kcnj6 |  | 2.05 | anxiety, depression |
| 181 | Keap1 | 8.55 | 1.94 | anxiety, depression |
| 182 | Kif1b |  | 0.57 | pain, anxiety, depression |
| 183 | Klf10 |  | 1.75 | anxiety, depression |
| 184 | Klf5 |  | 1.78 | anxiety, depression |
| 185 | Kmt2a | 1.86 |  | pain, depression |
| 186 | Kng1 |  | 0.13 | pain, anxiety, depression |
| 187 | Krt18 | 27.13 |  | anxiety, depression |
| 188 | Lbp | 2.71 |  | pain, anxiety, depression |
| 189 | Ldha |  | 17.95 | pain, anxiety, depression |
| 190 | Lef1 |  | 1.79 | anxiety, depression |
| 191 | Lgals1 |  | 1.78 | pain, anxiety, depression |
| 192 | Lipe |  | 0.18 | pain, anxiety, depression |
| 193 | Litaf | 5.02 |  | pain, anxiety, depression |
| 194 | Lpin1 | 1.95 |  | anxiety, depression |
| 195 | Lpl | 2.31 |  | pain, anxiety, depression |
| 196 | Maff | 2.08 |  | pain, anxiety, depression |
| 197 | Magi1 | 0.37 | 1.88 | anxiety, depression |
| 198 | Map2 | 34.82 | 1.60 | anxiety, depression |
| 199 | Map2k6 |  | 1.74 | pain, anxiety, depression |
| 200 | Mapk1 | 1.94 | 1.77 | pain, anxiety, depression |
| 201 | Mapk10 | 0.07 |  | anxiety, depression |
| 202 | Mapk14 |  | 1.75 | pain, anxiety, depression |
| 203 | Mapk3 | 2.72 |  | pain, anxiety, depression |
| 204 | Mapk8 | 0.10 |  | pain, anxiety, depression |
| 205 | Mapk9 | 1.85 | 1.68 | pain, anxiety, depression |
| 206 | Mbp | 0.19 |  | anxiety, depression |
| 207 | Mdm2 | 0.43 |  | pain, anxiety, depression |
| 208 | Mef2c | 0.06 | 4.28 | anxiety, depression |
| 209 | Men1 | 18.17 | 4.30 | pain, anxiety |
| 210 | Mgp |  | 2.60 | anxiety, depression |
| 211 | Mme | 4.03 |  | pain, anxiety, depression |
| 212 | Mogat1 | 4.36 |  | anxiety, depression |
| 213 | Mtm1 | 0.24 | 0.43 | pain, anxiety |
| 214 | Myc | 0.24 | 2.47 | pain, anxiety, depression |
| 215 | Myh7 | 1.83 |  | pain, anxiety, depression |
| 216 | Myof |  | 4.57 | anxiety, depression |
| 217 | Nabp1 |  | 3.61 | anxiety, depression |
| 218 | Ncf1 |  | 0.11 | pain, anxiety, depression |
| 219 | Nedd4l | 0.30 | 0.33 | anxiety, depression |
| 220 | Nedd9 | 0.43 |  | anxiety, depression |
| 221 | Nes | 0.40 |  | pain, anxiety, depression |
| 222 | Nfat5 | 0.32 |  | pain, anxiety |
| 223 | Nfia | 0.19 |  | anxiety, depression |
| 224 | Nfix | 0.34 |  | anxiety, depression |
| 225 | Nfkb1 | 0.28 |  | anxiety, depression |
| 226 | Nos1 | 9.87 | 0.53 | pain, anxiety, depression |
| 227 | Nr1h3 |  | 2.30 | pain, depression |
| 228 | Nr1i2 |  | 0.35 | pain, anxiety, depression |
| 229 | Nr1i3 |  | 0.09 | pain, anxiety, depression |
| 230 | Nr3c1 | 0.38 | 3.96 | pain, anxiety, depression |
| 231 | Nr3c2 | 0.62 |  | pain, anxiety, depression |
| 232 | Nr4a2 | 2.08 | 4.80 | anxiety, depression |
| 233 | Nr4a3 | 4.32 | 2.56 | anxiety, depression |
| 234 | Nrep | 0.55 |  | pain, anxiety, depression |
| 235 | Nrg1 | 0.40 |  | anxiety, depression |
| 236 | Nrxn3 | 0.26 |  | anxiety, depression |
| 237 | Nsd2 | 0.55 |  | anxiety, depression |
| 238 | Nt5e | 2.25 |  | pain, anxiety, depression |
| 239 | Nts |  | 0.88 | pain, anxiety, depression |
| 240 | Oprk1 | 7.00 | 2.83 | pain, anxiety |
| 241 | Oprl1 | 11.3 | 3.45 | pain, anxiety |
| 242 | Orm1 |  | 0.16 | pain, anxiety, depression |
| 243 | Osbpl1a | 0.66 |  | anxiety, depression |
| 244 | P2rx4 | 0.34 |  | pain, depression |
| 245 | P4ha1 |  | 3.17 | pain, anxiety, depression |
| 246 | Pcsk9 | 2.51 |  | pain, anxiety, depression |
| 247 | Pde4b | 1.81 | 1.66 | pain, anxiety, depression |
| 248 | Pde5a | 0.48 |  | anxiety, depression |
| 249 | Pde7b | 6.39 | 2.44 | anxiety, depression |
| 250 | Pdyn | 1.97 |  | pain, anxiety, depression |
| 251 | Penk | 2.94 |  | pain, anxiety, depression |
| 252 | Per1 |  | 3.25 | pain, anxiety, depression |
| 253 | Pfkfb3 | 0.11 |  | anxiety, depression |
| 254 | Pfkp |  | 1.81 | anxiety, depression |
| 255 | Pkp2 | 1.89 |  | anxiety, depression |
| 256 | Plaur | 3.31 |  | pain, depression |
| 257 | Plec | 0.16 | 8.18 | anxiety, depression |
| 258 | Plekhb1 |  | 0.52 | anxiety, depression |
| 259 | Pln | 0.37 | 0.11 | pain, anxiety |
| 260 | Pltp |  | 0.52 | anxiety, depression |
| 261 | Pnoc | 0.56 |  | pain, anxiety |
| 262 | Postn | 5.25 | 0.39 | pain, depression |
| 263 | Ppfibp1 | 1.58 | 2.07 | anxiety, depression |
| 264 | Ppp1r1b | 3.54 |  | pain, anxiety, depression |
| 265 | Prc1 | 10.25 |  | anxiety, depression |
| 266 | Prkar1a | 0.55 | 1.53 | pain, anxiety, depression |
| 267 | Prkcd |  | 0.11 | pain, anxiety, depression |
| 268 | Prkcz | 1.85 |  | pain, anxiety, depression |
| 269 | Prom1 |  | 0.27 | anxiety, depression |
| 270 | Prrt2 | 0.64 |  | pain, anxiety |
| 271 | Psen1 |  | 2.45 | pain, anxiety, depression |
| 272 | Psrc1 | 4.11 | 2.72 | anxiety, depression |
| 273 | Pth1r | 1.78 |  | pain, depression |
| 274 | Pttg1 |  | 4.61 | anxiety, depression |
| 275 | Pvalb | 0.66 |  | anxiety, depression |
| 276 | Pygl | 2.30 |  | pain, depression |
| 277 | Rac1 | 0.29 |  | pain, anxiety, depression |
| 278 | Racgap1 | 0.063 |  | anxiety, depression |
| 279 | Rai1 | 0.26 | 3.55 | anxiety, depression |
| 280 | Rassf4 | 4.17 |  | anxiety, depression |
| 281 | Rbms1 | 2.17 |  | anxiety, depression |
| 282 | Rbp4 |  | 36.55 | anxiety, depression |
| 283 | Retn | 3.56 |  | anxiety, depression |
| 284 | Rock2 | 2.36 |  | anxiety, depression |
| 285 | Runx1 | 10.06 |  | pain, depression |
| 286 | Runx2 | 4.29 |  | pain, anxiety, depression |
| 287 | Scg2 |  | 1.91 | anxiety, depression |
| 288 | Scn1a |  | 0.22 | pain, anxiety, depression |
| 289 | Scn1b | 0.64 |  | pain, anxiety, |
| 290 | Scn8a |  | 0.44 | pain, anxiety, depression |
| 291 | Serpine1 | 2.0 |  | pain, anxiety, depression |
| 292 | Set | 2.53 |  | anxiety, depression |
| 293 | Setd5 | 3.70 |  | anxiety, depression |
| 294 | Sgk1 | 0.29 |  | pain, anxiety, depression |
| 295 | Slc16a3 |  | 16.20 | anxiety, depression |
| 296 | Slc17a5 | 0.52 |  | pain, depression |
| 297 | Slc1a2 |  | 0.26 | pain, anxiety, depression |
| 298 | Slc22a6 |  | 3.52 | anxiety, depression |
| 299 | Slc22a7 | 0.14 |  | pain, depression |
| 300 | Slc29a1 | 47.32 | 0.43 | anxiety, depression |
| 301 | Socs2 | 0.27 |  | anxiety, depression |
| 302 | Socs3 |  | 1.91 | pain, anxiety, depression |
| 303 | Sorbs1 |  | 0.38 | anxiety, depression |
| 304 | Sparc | 3.05 | 3.44 | pain, anxiety, depression |
| 305 | Spata13 | 3.28 |  | anxiety, depression |
| 306 | Spp1 | 6.13 |  | pain, anxiety, depression |
| 307 | Stxbp1 |  | 2.05 | pain, depression |
| 308 | Sulf2 | 0.63 |  | anxiety, depression |
| 309 | Tac1 | 3.90 | 1.51 | pain, anxiety, depression |
| 310 | Tardbp | 0.42 | 20.0 | pain, anxiety, depression |
| 311 | Tcf4 | 0.35 | 0.14 | pain, depression |
| 312 | Tcf7l2 | 66.65 | 0.51 | pain, depression |
| 313 | Tek |  | 0.56 | pain, depression |
| 314 | Tmem132d |  | 1.98 | anxiety, depression |
| 315 | Tnc | 2.11 |  | pain, depression |
| 316 | Tnf |  | 5.88 | pain, anxiety, depression |
| 317 | Tph2 | 0.55 |  | anxiety, depression |
| 318 | Tpm1 | 4.89 | 1.55 | pain, anxiety, depression |
| 319 | Tpm3 | 3.37 | 2.14 | anxiety, depression |
| 320 | Trh | 3.67 |  | pain, anxiety, depression |
| 321 | Trp53 | 4.06 | 2.69 | pain, anxiety, depression |
| 322 | Tsc22d1 |  | 1.87 | anxiety, depression |
| 323 | Tsc22d3 |  | 2.10 | pain, anxiety, depression |
| 324 | Ttr | 115.89 |  | pain, anxiety, depression |
| 325 | Tuba8 |  | 2.0 | anxiety, depression |
| 326 | Txnip | 1.86 | 1.73 | pain, anxiety, depression |
| 327 | Ube2c | 4.97 | 7.50 | anxiety, depression |
| 328 | Ube3a |  | 3.77 | pain, anxiety, depression |
| 329 | Uhrf1 | 2.29 |  | anxiety, depression |
| 330 | Ulk1 |  | 9.10 | anxiety, depression |
| 331 | Upp1 |  | 1.92 | pain, anxiety, depression |
| 332 | Usp8 | 0.23 |  | pain, anxiety |
| 333 | Vgf |  | 2.17 | pain, anxiety, depression |
| 334 | Vim |  | 1.92 | pain, anxiety, depression |
| 335 | Vldlr |  | 0.51 | pain, anxiety, depression |
| 336 | Vps13a | 0.49 |  | pain, anxiety |
| 337 | Xiap | 3.57 | 0.25 | pain, anxiety, depression |
| 338 | Zbtb20 | 2.18 |  | anxiety, depression |
|  |  |  |  |  |
|  |  |  |  |  |
