# Supplementary figures and images for: Gene Transcript Alterations in the Spinal Cord, Anterior Cingulate Cortex, and Amygdala in Mice Following Peripheral Nerve Injury
Source: Front Cell Dev Biol. 2021 Apr 7;9:634810. doi: 10.3389/fcell.2021.634810 (PMC8059771; doi:10.3389/fcell.2021.634810)

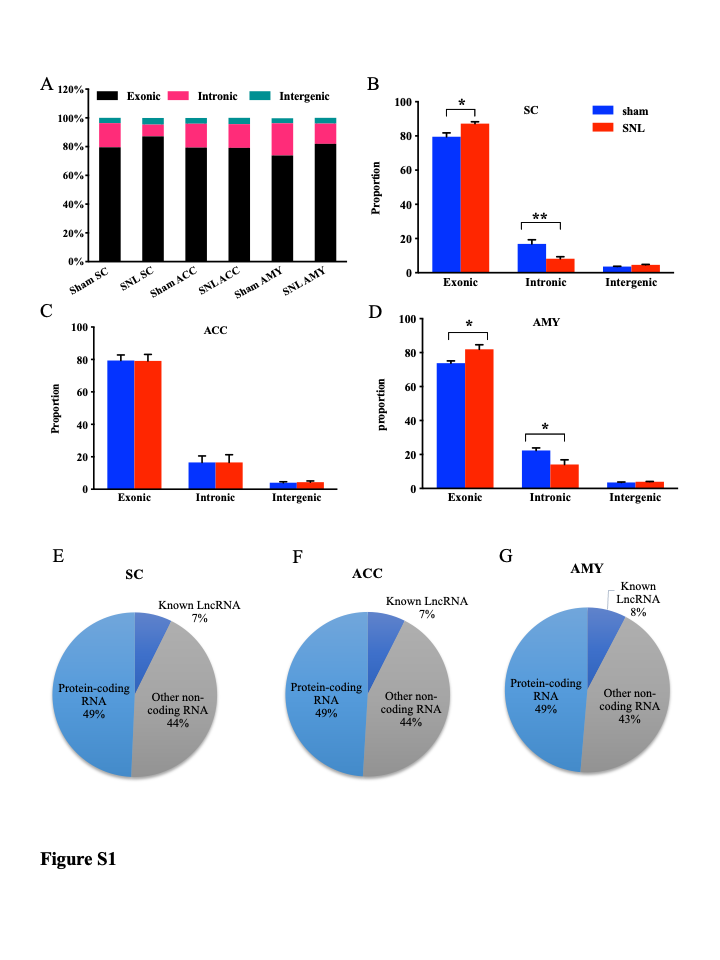

Supplement: Supplementary Figure 1 — Transcriptome profiling in the SC, ACC and AMY in mice after nerve injury. (A) The mapped proportions of exonic, intronic, and intergenic reads in the SC, ACC and AMY on day 7 post-SNL or sham surgery. (B-D) The proportions of reads that align to exonic, intronic and intergenic regions from the SC (B), ACC (C), and AMY (D) on day 7 post-surgery. n = 3 biological repeats. ∗P < 0.05; ∗∗P < 0.01 versus the corresponding sham group by two-tailed unpaired Student’s t-test. (E-G) Distribution of differentially expressed RNAs in SC (E), ACC (F), and AMY (G) on day 6 after SNL. [file Image_1.TIFF]

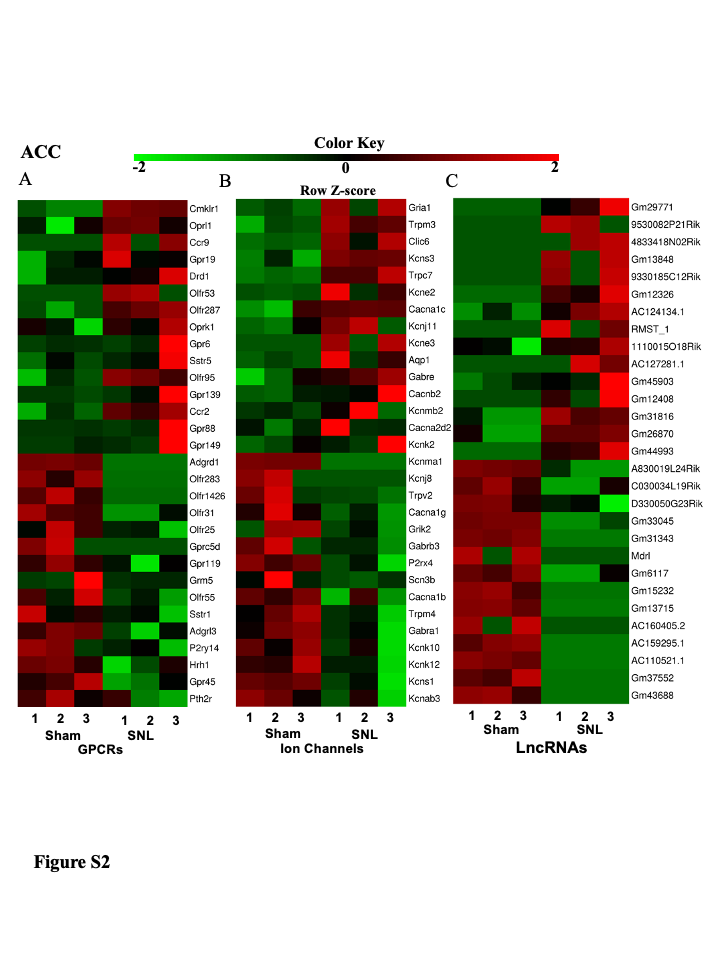

Supplement: Supplementary Figure 2 — Heatmaps of the representative differentially expressed genes (DEGs) in the ACC after nerve injury. Top 15 up-and down-regulated G protein-coupled receptor mRNAs (A), ion channel mRNAs (B), and lncRNAs (C) in the ACC on day 7 after SNL. Colors in the heatmaps indicate the Row Z-score among the different datasets. The up- and down-regulated genes are colored in red and green, respectively. [file Image_2.TIFF]

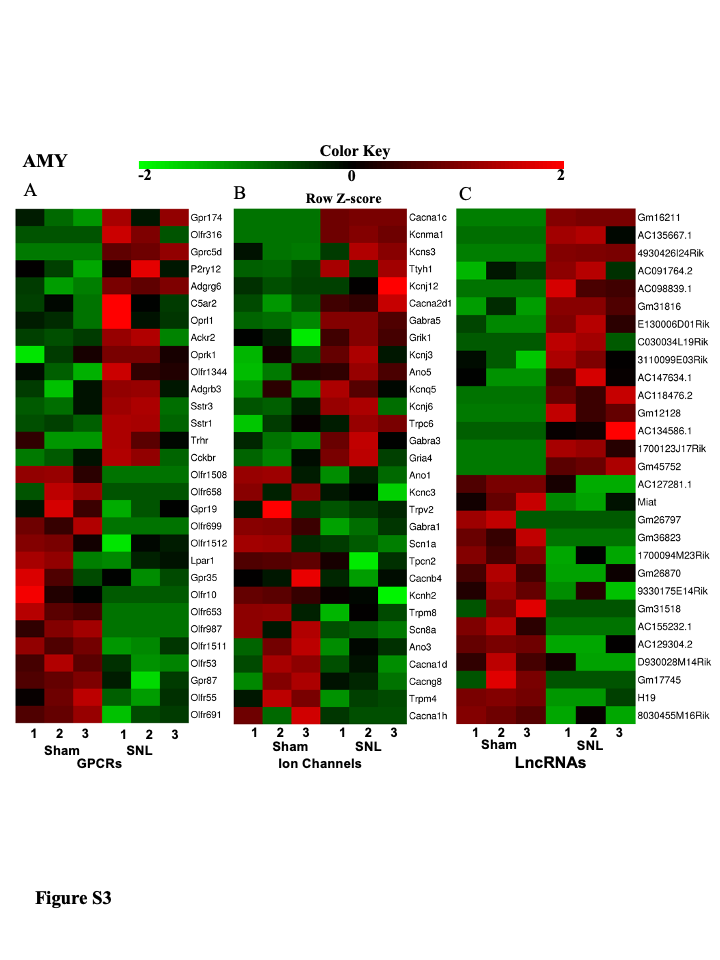

Supplement: Supplementary Figure 3 — Heatmaps of the representative differentially expressed genes (DEGs) in the AMY after nerve injury. Top 15 up- and down-regulated G protein-coupled receptor mRNAs (A), ion channel mRNAs (B), and lncRNAs (C) in the AMY on day 7 after SNL. Colors in the heatmaps indicate the Row Z-score among the different datasets. The up- and down-regulated genes are colored in red and green, respectively. [file Image_3.TIFF]

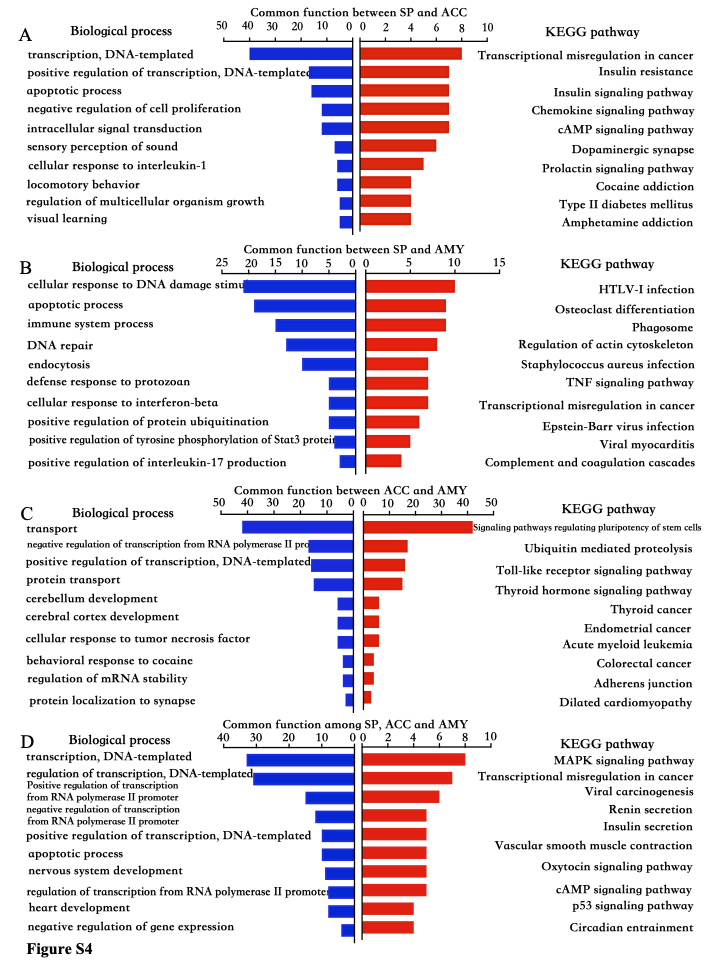

Supplement: Supplementary Figure 4 — The common biological processes and KEGG pathways in the SC, ACC and AMY after nerve injury. (A-D) The top 10 common enrichments in biological processes (blue panels on the left) and KEGG pathways (red panels on the right) between SC and ACC (A), SC and AMY (B), or ACC and AMY (C). The top 10 common function enrichments in biological processes (blue panels on the left) and KEGG pathways (red panels on the right) among SC, ACC and AMY (D) according to the P-value. Blue and red bars represent the biological process and pathway enrichments, respectively. [file Image_4.TIFF]

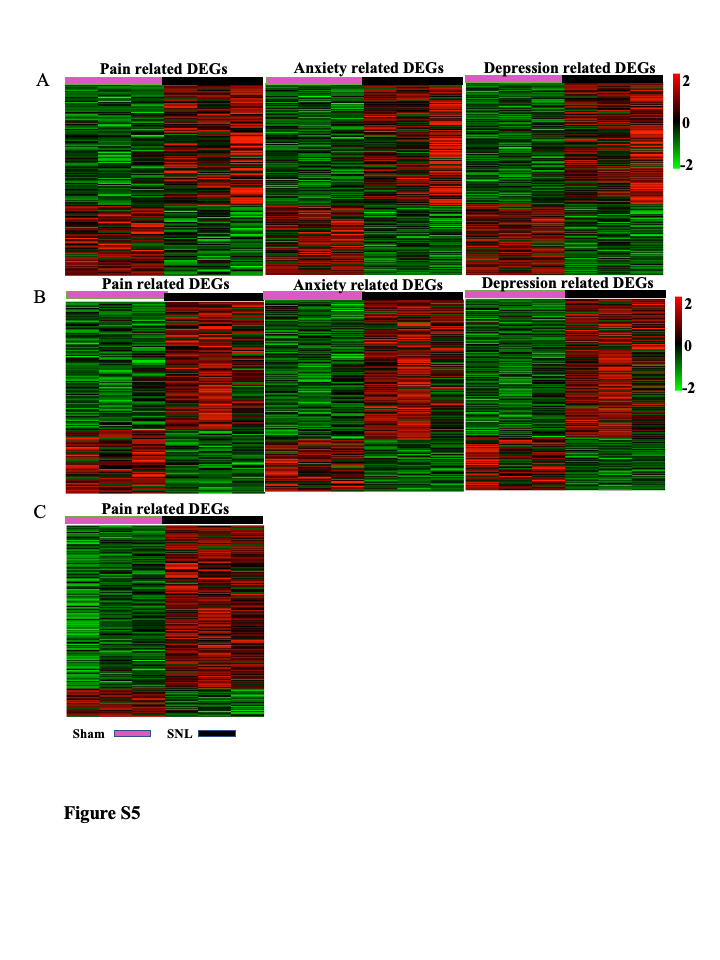

Supplement: Supplementary Figure 5 — Heatmaps of the identified pain-, anxiety- and depression-related genes in the SC, ACC and AMY after nerve injury. (A) Heatmaps of 157 (100 upregulated and 57 downregulated) pain-, 220 (140 upregulated and 80 downregulated) anxiety- and 278 (176 upregulated and 102 downregulated) depression-related DEGs in the ACC. (B) Heatmaps of 149 (100 upregulated and 49 downregulated) pain-, 201 (146 upregulated and 55 downregulated) anxiety- and 287 (207 upregulated and 80 downregulated) depression-related DEGs in the AMY; C, Heatmaps of 230 (196 upregulated and 34 downregulated) pain-related DEGs in the spinal cord. Colors in the heatmaps indicate the Row Z-score among the different datasets. The up- and down-regulated genes are colored in red and green, respectively. [file Image_5.TIFF]

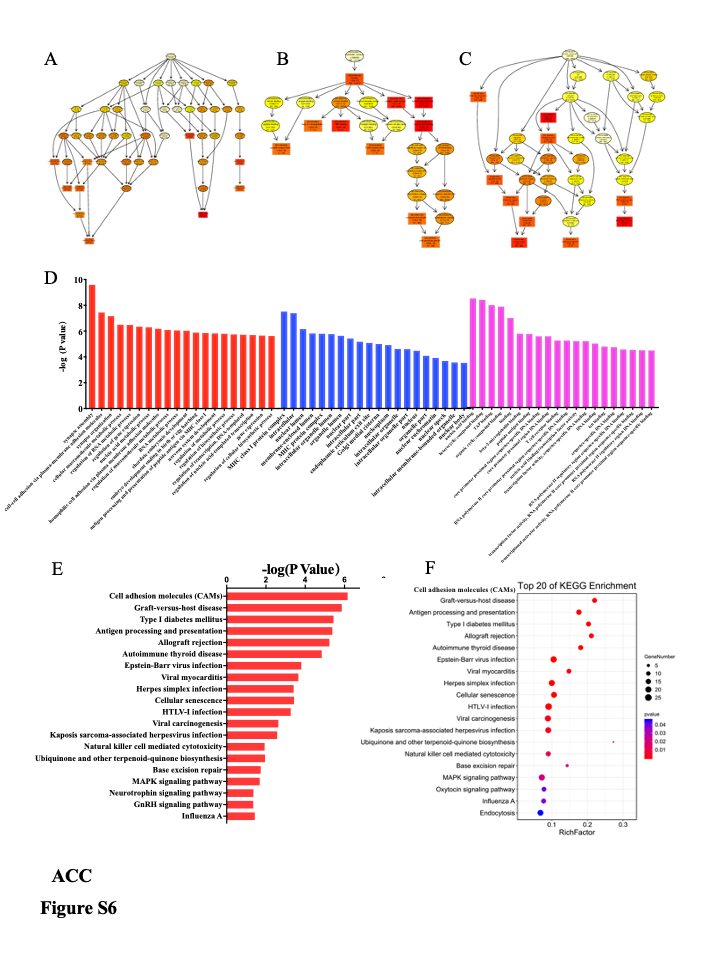

Supplement: Supplementary Figure 6 — The functional prediction of DE lncRNAs by GO and KEGG analyses in the ACC from SNL mice. (A-C) Directed Acyclic Graphs (DAGs) graphically display the significant GO enrichment results with the candidate targeted genes in biological process (A), molecular function (B), and cellular component (C). (D) The significant molecular function, biological process and cellular component enrichment analysis of DE lncRNA-related mRNAs. (E,F) The DE lncRNA-related mRNAs enriched KEGG pathways represented by enrichment scores (−log10 (P-value)) (E) and the scatterplot showing statistics of pathway enrichment (F), respectively. The color of pathway terms is defined by the enrichment P-value. [file Image_6.TIFF]

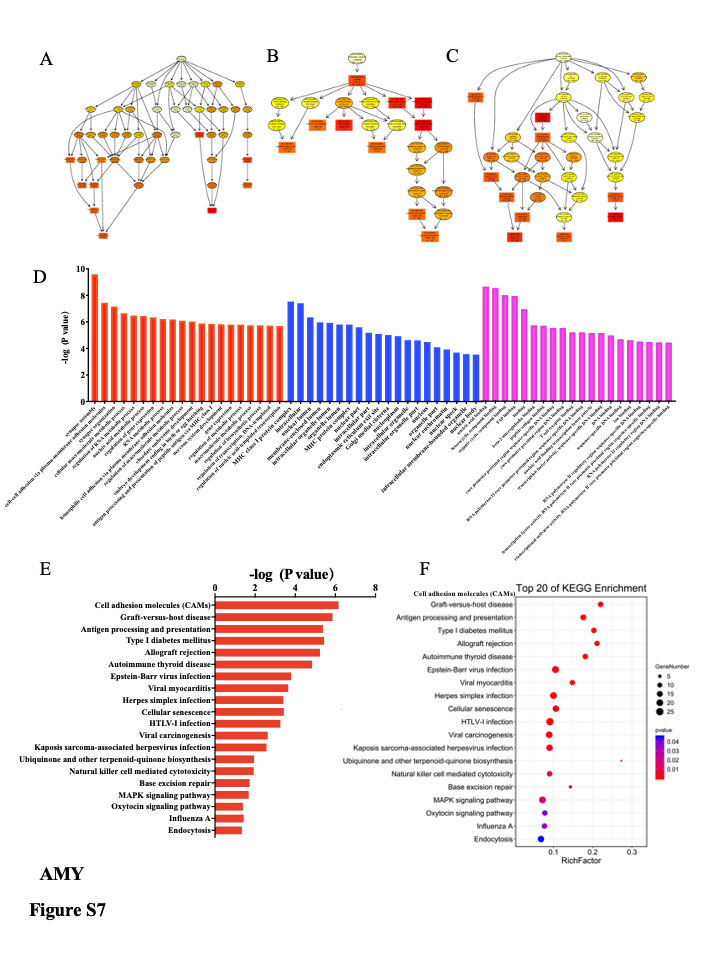

Supplement: Supplementary Figure 7 — The functional prediction of DE lncRNAs by GO and KEGG analyses in the AMY from SNL mice. (A-C) Directed Acyclic Graphs (DAGs) graphically display the significant GO enrichment results with the candidate targeted genes in biological process (A), molecular function (B), and cellular component (C). (D) The significant molecular function, biological process and cellular component enrichment analysis of DE lncRNA-related mRNAs. (E,F) The DE lncRNA-related mRNAs enriched KEGG pathways represented by enrichment scores (−log10 (P-value)) (E) and the scatterplot showing statistics of pathway enrichment (F), respectively. The color of pathway terms is defined by the enrichment P-value. [file Image_7.TIFF]
